# Characterization of the MYB Genes Reveals Insights Into Their Evolutionary Conservation, Structural Diversity, and Functional Roles in *Magnaporthe oryzae*

**DOI:** 10.3389/fmicb.2021.721530

**Published:** 2021-11-26

**Authors:** Sehee Lee, Ronny Völz, Hyeunjeong Song, William Harris, Yong-Hwan Lee

**Affiliations:** ^1^Department of Agricultural Biotechnology, Seoul National University, Seoul, South Korea; ^2^Interdisciplinary Program in Agricultural Genomics, Seoul National University, Seoul, South Korea; ^3^Center for Fungal Genetic Resources, Seoul National University, Seoul, South Korea; ^4^Plant Immunity Research Center, Seoul National University, Seoul, South Korea; ^5^Research Institute of Agriculture and Life Sciences, Seoul National University, Seoul, South Korea

**Keywords:** appressorium formation, host-plant recognition, cell wall integrity, hydrophobicity, *Magnaporthe oryzae*, melanization, MYB transcription factors, rice blast fungus

## Abstract

The myeloblastosis (MYB) transcription factor family is evolutionarily conserved among plants, animals, and fungi, and contributes to their growth and development. We identified and analyzed 10 putative MYB genes in *Magnaporthe oryzae* (*MoMYB*) and determined their phylogenetic relationships, revealing high divergence and variability. Although MYB domains are generally defined by three tandem repeats, MoMYBs contain one or two weakly conserved repeats embedded in extensive disordered regions. We characterized the secondary domain organization, disordered segments, and functional contributions of each *MoMYB*. During infection, *MoMYB*s are distinctively expressed and can be subdivided into two clades of being either up- or down-regulated. Among these, *MoMYB1* and *MoMYB8* are up-regulated during infection and vegetative growth, respectively. We found MoMYB1 localized predominantly to the cytosol during the formation of infection structures. Δ*Momyb1* exhibited reduced virulence on intact rice leaves corresponding to the diminished ability to form hypha-driven appressorium (HDA). We discovered that MoMYB1 regulates HDA formation on hard, hydrophobic surfaces, whereas host surfaces partially restored HDA formation in Δ*Momyb1*. Lipid droplet accumulation in hyphal tips and expression of HDA-associated genes were strongly perturbed in Δ*Momyb1* indicating genetic interaction of MoMYB1 with downstream components critical to HDA formation. We also found that MoMYB8 is necessary for fungal growth, dark-induced melanization of hyphae, and involved in higher abiotic stress tolerance. Taken together, we revealed a multifaceted picture of the MoMYB family, wherein a low degree of conservation has led to the development of distinct structures and functions, ranging from fungal growth to virulence.

## Introduction

*Magnaporthe oryzae* is one of the most threatening plant pathogens to global food security, infecting major cereal crops including rice, wheat, and barley. Rice blast caused by *M. oryzae* is responsible for significant yield losses worldwide (Talbot, 2003; Nalley et al., 2016). Furthermore, wheat blast disease is considered to be an emerging global threat (Islam et al., 2016).

The rice blast disease cycle begins when conidia adhere to the plant surface. Environmental cues, such as surface hydrophobicity, moisture, and host-associated molecular patterns (HAMPs) (Taylor and Gallo, 2006), induce the formation of a specialized infection structure called an appressorium from germinated conidia or mycelia (Wilson and Talbot, 2009). The appressorium generates high turgor pressure (up to 8 MPa), enabling the fungus to physically penetrate the plant cell. Turgor pressure is generated through the accumulation of glycerol in mature appressoria and the subsequent movement of water (Foster et al., 2017). The cell wall of the appressorium is heavily melanized to handle the high turgor pressure. After plant cell penetration, the fungus forms a bulbous invasive hypha in the infected plant cell. After invasive growth, conidia are produced on lesions and then dispersed. The appressorium is not necessary for rice root infection due to the ability of *M. oryzae* to form a hyphopodium, a swollen hyphal structure, to penetrate the rice root (Tucker et al., 2010).

Signaling pathways in *M. oryzae* involving secondary messengers and regulatory proteins, such as cyclic adenosine monophosphate (cAMP), Ca^2+^, G proteins, and mitogen-activated protein kinases (MAPKs), have been studied to elucidate the regulation of infection-related differentiation and pathogenesis (Mitchell and Dean, 1995; Xu and Hamer, 1996; Liu and Dean, 1997; Xu et al., 1997; Jeon et al., 2008; Choi et al., 2009; Rho et al., 2009). The perception of HAMP is critical to appressorium formation (Liu et al., 2011; Anjago et al., 2018). In this context, cutinase-mediated degradation of the host plant cuticle into cutin monomers elicits the cAMP/protein kinase A (PKA) and stimulates the diacylglycerol (DAG)/protein kinase C (PKC) signaling cascades, which are important for surface recognition and germ tube–driven appressoria (GDA) formation in *M. oryzae*. The cutinase MoCut2 is an upstream activator of the cAMP/PKA and DAG/PKC signaling pathways, which direct appressorium emergence and infectious growth in *Magnaporthe grisea*. Cutin monomers, cAMP, 3-isobutyl-1-methylxanthine, and DAG restore the defects of the *cut2* mutant, indicating that CUT2 is required for surface sensing and full virulence (Skamnioti and Gurr, 2007). Surface recognition and host-surface penetration are among the most critical processes in plant infection (Liu et al., 2011). MAPK cascades consist of three sequentially activated kinase modules comprised of a MAPK kinase kinase, a MAPK kinase, and, eventually, a MAPK, which link upstream signals to downstream targets through phosphorylation (Pitzschke et al., 2009). In *M. oryzae*, the Pmk1 MAPK cascade involves the sensing of environmental cues and their conveyance from MEKK Mst11 to MEK Mst7 and then to Pmk1 (Liu et al., 2011). The upstream sensors MoMsb2 and MoSho1 are critical for the sensing of physical and chemical signals, such as surface hydrophobicity, hardness, and cutin monomers and waxes on the host surface, and the activation of the Pmk1 kinase cascade through phosphorylation. Mutants of *Pmk1*, *MoMsb2*, and *MoSho1* show deficiencies in appressorium formation and virulence (Liu et al., 2011). The zinc finger transcription activator MoMsn2 plays an essential role in appressorium development and enhances the pathogenicity of *M. oryzae* (Zhang et al., 2014). The putative Rho GTPase–activating protein MoLRG1 is required for conidiation and GDA emergence (Ye et al., 2014). The homeobox transcription factor MoHOX7 is essential for appressorium formation and pathogenicity in *M. oryzae*. The addition of appressorium-inducing chemicals, such as cAMP and 1,16-hexadecanediol, could not restore the appressorium formation defect in the *Mohox7* mutant on hydrophobic or hydrophilic surfaces, suggesting that MoHOX7 is a crucial regulator of appressorium morphogenesis (Kim et al., 2009). The histone demethylase MoJMJ1 is also involved in appressorium development, indicating the importance of histone demethylation through MoJMJ1 during the infection process. *M. oryzae* also causes foliar disease *via* the formation of hypha-driven appressorium (HDA). HDA originate at the tip of an hypha, rather than at the tips of the conidial germ tubes (Kim et al., 2009). Importantly, MoHOX7, MoJMJ1, MoMsn2, and MoLRG1 are required to drive appressorium formation from hyphae and germ tubes (Anjago et al., 2018).

Several families of transcription factors (TFs) have been studied to clarify their roles in the regulation of crucial processes including conidiation, germination, secondary metabolite synthesis, and infection-related development in numerous fungi (Latchman, 1997; Kasuga et al., 1999; Shelest, 2008). Myeloblastosis (MYB) proteins were first identified in the avian myeloblastosis virus and have since been identified in all eukaryotes. Some MYB TFs function as oncogenes in humans (Bergthold et al., 2014), and plant MYB TFs regulate developmental processes and defense responses (Yanhui et al., 2006; Dubos et al., 2010; Katiyar et al., 2012; Salih et al., 2016). In fungi and particularly *M. grisea*, the underlying molecular mechanisms and biological functions of the MYB TF family have been poorly characterized. Generally, MYB TFs possess a MYB DNA-binding domain (DBD/MYB domain) containing up to four imperfect repeats. Each repeat is composed of 50–55 residues that fold into three alpha helices, the second and third of which form a helix–turn–helix structure. The three helices of each MYB repeat comprise a hydrophobic core, which provides a scaffold for insertion of the third helix into the major groove of the DNA molecule. MYB TFs are subdivided based on the number of repeats in their DBDs. The majority of MYB TFs contain two MYB repeats, and are designated R2R3 MYB proteins based on their similarity to the second and third repeats in vertebrates. Plant R2R3 MYB TFs recognize *cis*-regulatory sequences assigned as MYB-core motifs [C/T]NGTT[G/T] and AC-rich elements (Dubos et al., 2010). Structural analysis of AtMYB66/WER showed that the third alpha-helices in both the R2 and R3 repeats of WER fit in the major groove of the DNA, thereby specifically recognizing the DNA motif 5′-AACNGC-3′ (Wang et al., 2020).

Beyond the MYB domain, MYB proteins are characterized by high variability in length and extensive disordered (non-MYB) regions. Non-MYB regions are characterized by greater sequence diversity than MYB domains, and are considered to be vital for the understanding of the high degrees of structural and functional diversity in this family (Millard et al., 2019). In *Fusarium graminearum* (*Fg*), MYB TFs have been reported to regulate secondary metabolite synthesis, environmental stress responses, and pathogenicity in Lin et al. (2011, 2012); Son et al. (2011); and Kim et al. (2014). In *Aspergillus nidulans* (*An*), MYBs regulate sexual and asexual development by contributing to conidiospore and asexual spore production (Arratia-Quijada et al., 2012). *MoMyb1* of the *M. oryzae* strain Guy11 is required for conidiogenesis and root infection (Dong et al., 2015).

We identified 10 *MoMYB* genes in *M. oryzae* and determined their phylogenetic relationships, structural organization, and functional motif conservation. MoMYB1 is required for the establishment of HDA infection structures following host plant recognition. We found that MoMYB8 is involved in cell-wall composition and melanization. We conclude that MoMYBs are pivotal regulators orchestrating fungal development necessary for full virulence and mycelial growth in *M. oryzae*.

## Materials and Methods

### Fungal Strains and Culture Conditions

*Magnaporthe oryzae* KJ201, a strain isolated from infected rice, was obtained from the Center for Fungal Genetic Resources (CFGR)^1^ at Seoul National University, Seoul, South Korea. For conidiation, KJ201 and the mutants derived from KJ201 were cultured on V8 juice agar (V8A; 80 ml of V8 juice, 310 μl of 10 N NaOH, and 15 g of agar per liter) at 25°C under continuous fluorescent light. Measurement of mycelial growth was performed using modified complete agar medium (TCM) and minimal media (MM) as previously described (Talbot et al., 1997). Genomic DNA and total RNAs were extracted from mycelia cultured in liquid CM (6 g of yeast extract, 6 g of casamino acids, and 10 g of sucrose per liter) for 3 days at 25°C on a shaker at 150 rpm. Total RNAs were also extracted from cultures in liquid CM after treating them with stress agents (Congo Red, 300 ppm; Calcofluor White, 200 ppm; NaCl, 0.5 M; CuSO_4_, 2 M; MgCl_2_, 0.1 M) for 4 h at 25°C on a shaker at 150 rpm.

### Mining of the Genes Encoding MYB Transcription Factor

The *MYB* TF genes in *M. oryzae* were identified using InterPro 58.0 based on the Myb (IPR017930) and Myb-like (IPR017877) domains (Finn et al., 2017). The orthologs of each gene in other fungi were identified using Fungal Genome GOLD Standard on the Comparative Fungal Genomics Platform (CFGP)^2^ (Choi et al., 2012) and BLASTP with > 30% sequence identity and > 50% query coverage (*E*-value ≤ 1e-5) (Johnson et al., 2008).

### Phylogenetic Analysis

Sequence alignment of fungal MYB TFs was performed using CLUSTAL W 2.1 (Larkin et al., 2007). After sequence trimming using TrimAl v1.2 (Capella-Gutiérrez et al., 2009), the maximum likelihood method and neighbor-joining method with 1,000 bootstrap repetitions in RAxML 8.2.9 (Stamatakis, 2014) and MEGA 7 (Kumar et al., 2016) were applied, respectively. The phylogenetic trees were modified using MEGA 7.

### Protoplast Production

After culturing mycelia in liquid CM in a shaker set at 150 rpm for 3 days, they were harvested by centrifugation for 10 min at 5,000 rpm, washed twice with SDW, and resuspended in 20% sucrose. Lysing enzyme (Sigma-Aldrich, St. Louis, MO, United States) was added at the final concentration of 2 mg/ml. Samples were taken every half hour to check the progress of protoplast generation using a light microscope and INCYTO^TM^ C-Chip^TM^ Disposable Hemocytometers as previously described (Jeon et al., 2008). To separate protoplasts from mycelia, sterile Miracloth was used.

### Generation of Deletion Mutants in Individual MoMYB Transcription Factor Genes and Complementation

Individual genes were deleted *via* homologous recombination using gene-deletion constructs created using a double-joint PCR method (Yu et al., 2004). A hygromycin B phosphotransferase gene cassette (HPH) derived from pBCATPH was used for selecting transformants (Chung et al., 2013). Each mutant allele was introduced into KJ201 protoplasts as previously reported (Liu and Friesen, 2012). Transformants were selected on TB3 agar medium (20% sucrose, 1% glucose, 0.3% yeast extract, 0.3% casamino acids, and 0.8% agar) supplemented with hygromycin B (200 ppm in final concentration). Genomic DNA was extracted from individual transformants using a previously reported method (Chi et al., 2009). They were analyzed by PCR in a C1000 thermal cycler (Bio-Rad, Hercules, CA, United States) to confirm gene deletion. This PCR reaction included 1 μl of qRTF and qRTR primers for each gene (100 nM for each primer), 5 μg of 2x PCR Master mix solution (i-StarMAX II) containing dNTPs, PCR buffer, i-StarMAX DNA polymerase, and loading dye (iNtRON Biotechnology, Seongnam, South Korea).

Complemented strains for selected *MoMYB* TF mutants were generated by co-transforming each gene construct and pII99, a vector containing the geneticin-resistant gene as a selection marker. Each gene construct consisted of the ORF and its 5′- and 3′- flanking regions. Resulting transformants were selected using TB3 agar medium supplemented with geneticin (800 ppm). All primers used in this study are listed in Supplementary Table 4. All strains produced in this study were deposited into CFGR.

### Southern Analysis

Genomic DNA extraction from mycelia, restriction enzyme digestion, agarose gel electrophoresis, and hybridization were performed following the standard procedures (Russell and Sambrook, 2001). A 5′- or 3′-flanking region of each mutagenized gene was used as a probe. Probe labeling with ^32^P was performed using Rediprime II Random Prime^TM^ Labeling System kit (Amersham Pharmacia Biotech, Piscataway, NJ, United States) according to the manufacturer’s instructions.

### Rice Infection Assays

Conidia collected from 7-day-old culture on V8A were used to infect rice leaves and sheath. Harvested conidia suspended in 250 ppm Tween 20 (10^5^ conidia/ml) were sprayed onto *Oryza sativa* cv. Nakdongbyeo, a cultivar susceptible to KJ201, at the three- or four-leaf stage. The inoculated seedlings were incubated in a dew chamber at 25°C for 24 h under dark and subsequently moved to a growth chamber set at 28°C, 80% humidity, and a photoperiod of 16 h with fluorescent light (Valent and Chumley, 1991). At 7 days after inoculation, rice leaves with blast lesions were evaluated using a previously used disease score system (Valent et al., 1991). We also infected intact and wounded leaves by placing culture blocks on MM (5 mm in diameter). Inoculated plants were placed in a moist chamber at 25°C for 5–7 days. Root infection was performed as previously described (Tucker et al., 2010). Two surface-sterilized rice seeds per pot were planted in sterilized vermiculite, and their roots were infected by incorporating four mycelial agar blocks per pot. All inoculated plants were incubated at 28°C for 2 weeks under the 12/12 h light/dark cycle. For sheath inoculation, a conidial suspension (2 × 10^4^ conidia/μl) was injected into excised rice sheaths. Inoculated samples were incubated in a moistened container at 25°C for 48 h (Koga et al., 2004). The chlorophyll enriched parts were removed using a razor blade. Epidermal layers with approximately three cell layers thick were observed using a light microscope to assess fungal proliferation in infected cells.

### Evaluation of Growth and Developmental Characteristics

All mutants were evaluated for mycelial growth, conidiation, conidial germination, and appressorium formation. Mycelial growth was measured using both TCM and with a stress agent. A culture plug (5 mm in diameter) on MM was placed on the center of each plate, and the inoculated plates were placed in an incubator at 25°C and constant fluorescent light, constant dark in case of pigmentation test, for 9 or 10 days.

Conidiation, conidial germination, and appressorium formation were evaluated as follows. Conidia produced by individual strains were harvested from 7-day-old V8A cultures using 5 ml sterilized distilled water (SDW) per plate and counted using INCYTO^TM^ C-Chip^TM^ Disposable Hemacytometers (Thermo Fisher Scientific, San Jose, CA, United States). Conidial germination and appressorium formation were examined at multiple time points. Harvested conidia were passed through one layer of Miracloth (CalBiochem, San Diego, CA, United States). After adjusting the concentration to 5 × 10^4^ conidia/ml, 70 μl conidial suspension was dropped on each hydrophobic microscope coverslip and kept in a moistened container at 25°C. Germinated conidia at 2 h post inoculation (hpi) and those that formed the appressorium (counted at 8 hpi) were counted using three replicates in three independent experiments (*n* ≥ 100). For checking the formation of hypha-driven appressorium (HDA), blocks of culture on MM were placed on the coverslip and incubated in a moistened container at 25°C with constant fluorescent light. After 24, 48, and 72 hpi, the agar blocks were removed, and the formation of HDA was checked using a light microscope.

### Evaluation of Surface Hydrophobicity

All strains used in this assay were cultured on oatmeal agar (OMA; 50 g of oatmeal and 25 g of agar per liter) at 25°C under constant fluorescent light until they sporulate. Drops (10 μl per drop) of SDW and 0.02% SDS in 50 mM EDTA were placed on the surface of individual cultures and observe them after 5 min as previously described (Kim et al., 2005).

### Gene Expression Analysis

Total RNAs were extracted from frozen mycelia using the Easy-Spin total RNA extraction kit (iNtRON Biotechnology, Seongnam, South Korea) according to the manufacturer’s instruction. 5 μg of total RNAs were reverse transcribed using oligo dT primer and ImProm-II^TM^ Reverse Transcription System (Promega, Madison, WI, United States). Each quantitative real-time PCR (qRT-PCR) reaction was performed in 10 μl solution that contains 2 μl of cDNA template (12.5 ng/μl), 3 μl of forward and reverse primers (100 nM for each primer) and 5 μl of 2x Rotor-Gene SYBR Green PCR Master Mix (Qiagen, Hilden, Germany). The cycling conditions were one cycle of 3 min denaturation at 95°C followed by 40 cycles of 15 s at 95°C, 30 s at 60°C and 30 s at 72°C in a Rotor-Gene Q 2plex (Qiagen, Hilden, Germany). The fold change of each gene using the average threshold cycle (Ct) was normalized using b-tubulin and calculated *via* the 2^–ΔΔCt^ method [ΔΔCt = (C_t, target gene_ − C_t, b–tubulin_)_treated_ − (C_t, target gene_ − C_t, b–tubulin_)_control_]. The primers used for qRT-PCR are listed in Supplementary Table 4. Gene expression in invasive hypha was visualized to heat map using Morpheus^3^.

### Histochemical Staining of H_2_O_2_

*In situ* detection of H_2_O_2_ was performed as previously described (Volz et al., 2021). In brief, four- week old rice plants were inoculated with *M. oryzae* mycelia agar block. After 6 days, the infected leaves were submerged in DAB solution (50 mg DAB, 130 mg Na_2_HPO_4_, 0.01%v/v Tween 20 in 50 ml H_2_O) and incubated for 6 h. Subsequently, the pistils were mounted in 20% glycerol and analyzed under a stereo-microscope.

## Results

### Identification of *Magnaporthe oryzae* Genes Encoding MYB Transcription Factors

To obtain insight into the MYB TF family of *M. oryzae*, we employed the InterPro analysis platform (Finn et al., 2017). We performed genome-wide screening of genes containing both the MYB (IPR017930) and MYB-like (IPR017877) domains, and determined their phylogenetic relationships. Our analysis revealed 10 MYB TFs in *M. oryzae* characterized by one or two inherent MYB-repeat domains. The 10 genomic loci encoding putative MoMYB TFs are distributed widely over 7 chromosomes (Supplementary Table 1).

**FIGURE 1:**
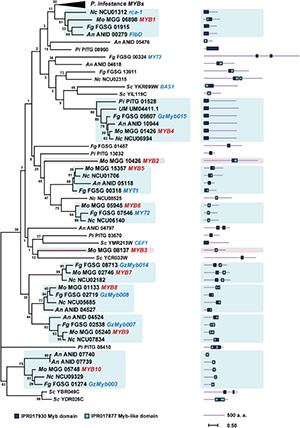
Phylogenetic tree of MYB TFs of six fungi and one oomycete generated using the maximum-likelihood method for 63 MYB TFs of the following species: *Magnaporthe oryzae* (*Mo*), *Aspergillus nidulans* (*An*), *Fusarium graminearum* (*Fg*), *Neurospora crassa* (*Nc*), *Phytophthora infestans* (*Pi*), *Saccharomyces cerevisiae* (*Sc*), and *Ustilago maydis* (*Um*). Orthologous clades containing MoMYBs are highlighted. Characterized MoMYBs and previously studied MYB TFs of other species are represented with red and blue letters, respectively. The protein length of each TF is shown next to the tree. Positions of the MYB and MYB-like domains are indicated with color-coded rectangles.

To elucidate their evolutionary conservation, we performed a phylogenetic analysis of the MoMYB TFs and their homologs in other fungal and protists species belonging to *Ascomycota, Basidiomycota*, and *Oomycota* (Figure 1 and Supplementary Table 2). We included *MYB* orthologs in *Ustilago maydis* (*Um*), *An*, *Fg, Saccharomyces cerevisiae* (*Sc*), *Neurospora crassa* (*Nc*), and *Phytophthora infestans* (*Pi*). We found that *MoMYB*s are distributed widely among the MYB orthologs of related species and do not cluster closely with each other. This finding indicates a diverse molecular composition in the *MoMYB* family, suggesting multiple underlying biological functions. For example, MoMYB4 was clustered with its orthologs in *Nc*, *An*, *Fg*, *Um*, and *Pi*, which consistently show strong conservation along the entire amino acid sequence, including in the N-terminal MYB domain and disordered C-terminus (Figure 1). Likewise, MoMYB1 and MoMYB5–MoMYB10 exhibited higher degrees of conservation with MYBs from *Fg*, *Nc*, and *An*. This finding indicates a common evolutionary origin of clustered MYBs, which supports the conservation of functions and the notion of strong sub-functionalization among MYB members in a single species.

### MYB Domain Structure

**FIGURE 2:**
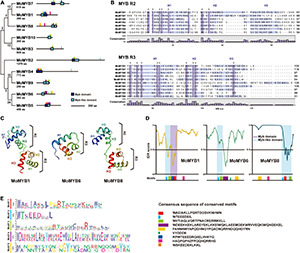
Genetic relationships and secondary structures of MoMYB TFs. **(A)** Genetic relationship and motif analyses of 10 MoMYB TFs were conducted using the neighbor-joining method and MEME, respectively. **(B)** R2-R3 repeat domain alignment of MoMYBs constructed using ClustalW. The dark-purple boxes and asterisks indicate conserved (≥60%) amino-acid sequences. Light-purple boxes indicate helix regions according to the DSSP algorithm (Kabsch and Sander, 1983). **(C)** Secondary structures of the MYB-related domains of MoMYB1, MoMYB3, and MoMYB8 predicted using SWISS-MODEL. **(D,E)** Disordered regions analyzed using PONDR (Garner et al., 1999) and predicted motifs are indicated below the graph. The disorder threshold was set to 0.5. Consensus motifs in panels **(A,D)** are presented in panel **(E)**. Motifs are color coded throughout each figure panel.

Phylogenetic analysis of 10 MoMYB gene’s proteins based on the amino acid sequences of the conserved MYB and MYB-like domains revealed a rather weak relationship among these genes (Figure 2A). Clustering of MYB TFs into subgroups revealed clades whose individual members might be involved in the same or similar biological functions. Although the protein sequence exhibited a low degree of conservation, we found stronger phylogenetic relationships between the gene pairs MoMYB1 and MoMYB7, MoMYB4 and MoMYB9, MoMYB5 and MoMYB6, and MoMYB3 and MoMYB10. On the other hand, MoMYB2 and MoMYB8 were not associated closely with other members. Sequence alignment indicated that the MYB repeats in *M*. *oryzae* correspond to the R2R3 MYB type in humans, *Drosophila* spp., plants, and oomycetes (Supplementary Figure 1). The MoMYB domain structure, comprised of three helices, was conserved among the MoMYBs investigated (Supplementary Figure 2). Notably, the alignments of R2 and R3 in MoMYBs revealed greater conservation of R3 than R2 (Figure 2B). Despite previous reports that MYBs are generally defined by three tandem repeated domains (Kasuga et al., 1999; Shelest, 2008; Dubos et al., 2010), we found that seven MoMYBs harbored two repeated MYB domains, whereas MoMYBs 3, 6, and 10 each possessed single domains corresponding to R3 (Supplementary Figure 2). Interestingly, the distinguishing tryptophans that characterize MYB domains generally exhibit a low degree of conservation in the R2 domain (Figure 2B), suggesting low selective pressure that has enabled sub-functionalization.

Prediction of the secondary structure of the R2R3 MYB type found in MoMYB1 and MoMYB8 revealed a rather well conserved scaffold (Figure 2C and Supplementary Figure 2), which might enable protein–DNA interactions, as shown previously for MYB orthologs (Waterhouse et al., 2018). However, the MYBs containing only R3 domains exhibited *bona fide* unstructured MYB domains, suggesting that their DNA-binding ability might be affected (Figure 2C and Supplementary Figure 2). In summary, the predicted secondary structures indicate that the majority of MoMYBs are transcriptional regulators, and suggest divergent evolutionary pressures on the DNA-binding capacity of MoMYBs, which may reflect distinct biological functions of various MoMYB members.

### Disordered and Non-MYB Regions in MYB Proteins

DNA-binding properties are very conserved and similar among MoMYB proteins, suggesting that non-MYB regions have differentiated traits and functions. Most MYB ortholog sequences in plants and animals exhibit typical domain organization, with the MYB domain located at the N-terminus (Millard et al., 2019). However, in fungi and particularly in *M. oryzae*, many MoMYBs (e.g., MoMYB2, MoMYB3, and MoMYB7) do not follow this prevalent structural organization and instead contain large disordered extensions between the DBDs and the N-terminus (Figures 1, 2A,D,E and Supplementary Figure 3). Only MoMYB4 and its orthologs in *Pi*, *Um*, *Fg*, *An*, and *Nc* maintain strict localization of the MYB domain at the N-terminus. Overall, the non-MYB regions possess extraordinary variability in length and position relative to the DBD. Regions of high conservation observed in the disordered sequences could be used to define several motifs in various subgroups of the MoMYB family (Figures 2A,D,E and Supplementary Figure 3). For example, the green motif was detected in MoMYB1, MoMYB6, and MoMYB8, and may contribute to R3 folding. Only MoMYB6 and MoMYB8 contained the yellow motif, at their C- and N-termini, respectively, and the magenta motif is unique to MoMYB1 and MoMYB8 (Figures 2D,E and Supplementary Figure 3). Although we identified several subgroup motifs, their molecular functions remain unknown and additional research is needed to establish their functional annotations and thereby clarify *M. oryzae* MYB diversity.

### Expression Patterns of *MoMYB* Genes During Host Infection

**FIGURE 3:**
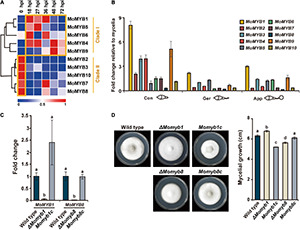
Expressions of *MoMYB*s during the *M. oryzae* life cycle were profiled and Δ*Momyb1* and Δ*Momyb8* deletion mutants were characterized. **(A)** Expression of *MoMYBs* during infection compared with mycelial cells [referred to as 0 h post infection (hpi)]. The *RNA*-seq dataset was published previously by Jeon et al. (2020). **(B)** Expression patterns of *MoMYB* TFs at various developmental stages. Con, conidia; Ger, germinating conidia and germ tube; App, conidia-forming appressoria (2–4 hpi). The fold-change values were calculated using the 2^–ΔΔCt^ method, and the β*-tubulin* gene and mycelial cDNA were used for normalization. **(C)** Validation of gene deletion in Δ*Momyb1* and Δ*Momyb8* knockout strains compared with the WT and *MYB* complementation strains (*Momyb1c* and *Momyb8c*). **(D)** Growth evaluation of the WT, Δ*Momyb1*, Δ*Momyb8*, *Momyb1c*, and *Momyb8c* grown on TCM for 9 days at 25°C. The diameter of each culture was measured. Statistical analysis using Tukey’s test was performed with three biological replicates (*p* < 0.05).

To obtain a comprehensive overview of *MoMYB* gene expression following rice infection, we analyzed expression patterns using a sheath infection and RNA sequencing approach (Jeon et al., 2020). The expression of *MoMYB* genes (Figure 3A) was analyzed at 0, 18, 27, 36, 48, and 72 h post infection (hpi), with 0 hpi being synonymous with uninfected vegetative mycelium. Based on their expression, the *MoMYB* genes could be grouped into two clades (Figure 3A). *MoMYB1*, *MoMYB4*, *MoMYB5*, *MoMYB6*, and *MoMYB9* were generally up-regulated after host infection and were assigned to clade I. Clade II contains *MoMYB2*, *MoMYB3*, *MoMYB10*, *MoMYB7*, and *MoMYB8*, which mainly showed strong expression in the vegetative mycelium and reduced expression after rice infection. *MoMYB7* and *MoMYB8* exhibited lesser expression shortly after infection and elevated expression at 36 hpi. These data suggest that *MoMYB*s play distinct roles during the infection process and that the control of their expression appears to be one element regulating their biological impacts.

Subsequently, we raised the question of which *MoMYB*s play dominant roles in the establishment of infection structures. Thus, we analyzed the expression of *MoMYB* genes during conidial development relative to that during vegetative mycelial growth. First, we detected increased expression of *MoMYB* genes in conidia. *MoMYB1* and *MoMYB9* showed more than fivefold increases, whereas the expression levels of *MoMYB5* and *MoMYB8* were only moderately elevated (Figure 3B). Second, we observed relatively low expression of *MoMYB* genes in germinating conidia, with the exception that *MoMYB1* showed a twofold increase relative to that in vegetative mycelia. The expression of *MoMYB*s was elevated in the appressorium, similar to the germinating conidia. Notably, *MoMYB1* showed the greatest transcript abundance of all *MoMYB* genes at various developmental stages, suggesting that it plays a dominant role in the control of *MYB*-associated functions, including conidial development and appressorium formation.

### Characterization of *MoMYB* Deletion Mutants

To determine whether individual MYB TFs participate in fungal development and pathogenicity, we generated target-gene deletion mutants for the majority of *MoMYB* genes by homologous recombination using the KJ201 *M. oryzae* strain. Deletion mutants were generated for eight of the 10 *MoMYB* genes (Supplementary Figure 4). A single insertion of the transgene was verified and confirmed through Southern hybridization. The lack of target gene expression was confirmed through quantitative reverse-transcription polymerase chain reaction (qRT-PCR) (Figure 3C and Supplementary Figure 4). Mycelial growth of all mutant strains was tested on TCM for 9 days (Figure 3D, Supplementary Figure 4, and Supplementary Table 3). Δ*Momyb1* showed a high growth rate, whereas Δ*Momyb8* exhibited reduced mycelial growth compared with the wild type (WT). To validate whether the compromised growth rate could be attributed to knockout of the *MoMYB*s, we introduced the corresponding complementation constructs to Δ*Momyb1* and Δ*Momyb8*. The complemented mutant strains *Momyb1c* and *Momyb8c* showed growth rates indistinguishable from that of the WT (Figure 3D). This result indicates that the effect on mycelial growth in Δ*Momyb1* and Δ*Momyb8* is caused by knockout of *MoMYB* genes.

To evaluate whether individual *MoMYB* deletions affect conidiation, germination, and appressorium formation, we analyzed all mutant strains on a hydrophobic surface (Supplementary Table 3). We confirmed the previously reported mycelial growth and conidiation defect (Dong et al., 2015) of Δ*Momyb1*, but detected no difference in conidiation, germination, or GDA formation in the other deletion mutants. This result highlights MoMYB1 as a pivotal factor governing infection-related processes in *M. oryzae*.

To assess the functions of MoMYBs in fungal virulence over the course of plant infection, we performed pathogenicity tests on rice leaves and roots using conidial suspensions and mycelial agar blocks of each deletion mutant (Supplementary Figure 5 and Supplementary Table 3). The infection level was indistinguishable between Δ*Momyb* mutant strains and the WT, indicating that knockout of individual *MoMYB*s did not interfere with fungal virulence and suggesting their functional redundancy in this process.

### MoMYB1 Conditionally Localizes to Different Cell Compartments

**FIGURE 4:**
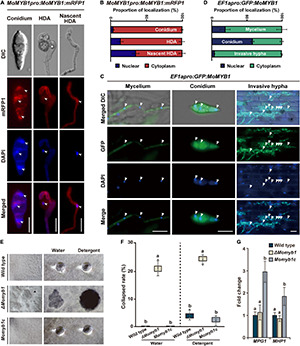
The structural integrity of aerial mycelia is compromised in Δ*Momyb1*. **(A–D)** Subcellular-localization study of MoMYB1 driven by the native promoter **(A,B)** and by a constitutively active promoter **(C,D)**. 4′,6-Diamidino-2-phenylindole (DAPI) was used for co-localization analysis in the nucleus. Scale bars represent 10 μm. Quantitative analysis of MoMYB1 localization is depicted in panels **(B,D)**. **(E,F)** Aerial mycelial hydrophobicity analysis was performed using the WT, Δ*Momyb1*, and *Momyb1c*. Water and a detergent (0.02% SDS) were dropped onto aerial mycelia and the degree of absorbance was monitored. **(G)** Expression patterns of the hydrophobicity-related genes *MPG1* and *MHP1* in the WT, Δ*Momyb1*, and *Momyb1c* are shown. Fold-change values and error bars (± SDs) were calculated using 2^–ΔΔCt^, and β*-tubulin* was used for normalization. Experiments were performed in three biological replicates. Significance was determined using Tukey’s test (*p* < 0.05).

To analyze the localization of MoMYB1 at various fungal development stages and throughout the course of rice infection, we expressed *MoMYB1* fused in-frame to RFP driven by its native promoter. In the conidia, MoMYB1 localized predominantly to the cytoplasm, in close vicinity to the nucleus, as demonstrated through nuclear staining with 4′,6-diamidino-2-phenylindole (DAPI) (Figures 4A,B). Likewise, MoMYB1 was largely found in the cytoplasm of HDA-forming hyphal tips (nascent HDA), but also accumulated in the nucleus in about 34% of analyzed samples. In the mature HDA, we detected MoMYB1:RFP in the cytoplasm and in approximately 15% of analyzed specimens, we spotted a strong signal in the nucleus These results suggest that MYB1 localizes in a developmental-dependent manner to the cytoplasm or nucleus.

To gain more insight into MoMYB1 localization, we expressed it under a strongly, ubiquitously active *EF1*α promoter from *Fusarium verticillioides.* Localization to the conidia was rather distinct to the line expressing *MoMYB1* by its native promotor. When expressed under the *EF1*α promoter, GFP:MoMYB1 was mainly detected in the nucleus, and was present in the surrounding cytoplasm in about 40% of samples (Figures 4C,D). The distinct nuclear localization of GFP-MYB1 driven by *EF1apro* might be attributed to the higher protein abundance facilitating the enrichment and detection in the nucleus. In the mycelium, we observed GFP:MoMYB1 mainly to the cytoplasm, and to 20% in the nucleus.

During the infection process, MoMYB1 is principally cytosolically localized in invasive hyphae (Figures 4C,D). Likewise, we observed cytoplasmic green fluorescent protein GFP:MoMYB1 in the GDA to the same extent as observed under the native promotor (Supplementary Figures 6A,B). During the establishment of distinct infection structures, such as the formation of HDA and in mature conidia, MoMYB1 strengthen a nuclear translocation, suggesting that it has regulatory functions during those processes that require specific subcellular localization patterns. Taken together, these findings demonstrate a conditional localization of MoMYB1 during *M. oryzae* development.

### MoMYB1 Determines the Structural Integrity of Aerial Mycelia

Cell wall hydrophobicity in fungi is necessary to reduce surface tension and allow mycelial growth into the aerial space. We found that the structural integrity of the aerial mycelia was affected in Δ*Momyb1*, resulting in a caved-in appearance of the aerial structure (Figure 4E). To determine their hydrophobicity, aerial mycelia were exposed to sterile water and a solution containing 0.02% sodium dodecyl sulfate (SDS). After application, the water and SDS drops could not be absorbed and remained on the upper aerial mycelia in *Momyb1c* and the WT (Figures 4E,F). In Δ*Momyb1*, however, the administered water and SDS-solution were absorbed by the mycelia, and no droplet was observed (Figures 4E,F). This finding indicates that the hydrophobicity and consequently the structural integrity of the aerial mycelia are perturbed in Δ*Momyb1*. This phenotype is reminiscent of the “easily wettable phenotype” described for the hydrophobin mutants Δ*mpg1* (Talbot et al., 1993) and Δ*mhp1* (Kim et al., 2005). To determine whether deletion of MoMYB1 interferes with the expression of these two genes, we analyzed their expression in Δ*Momyb1*. The expression of both genes in Δ*Momyb1* was indistinguishable from that in the WT (Figure 4G). However, we found that the transcript abundance of these genes is elevated in *Momyb1c*. This result suggests that MoMYB1 is their upstream regulator and that redundant MYB proteins compensate for the lack of MoMYB1 to enable hydrophobin expression. Furthermore, these findings suggest that MoMYB1 exerts transcriptional control over additional genes associated with cell wall composition and the establishment of aerial structures.

### MoMYB1 Is Necessary for Hypha-Driven Appressorium Formation and Orchestrates Genes Involved in This Process

**FIGURE 5:**
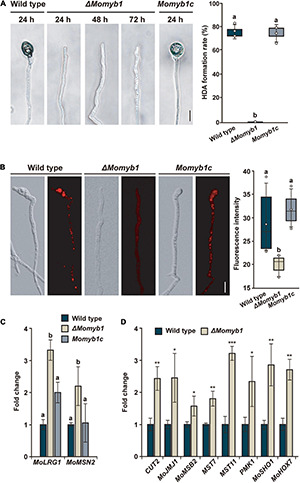
MoMYB1 is necessary for HDA formation and fine-tunes factors that govern this process. **(A)** HDA formation was determined after placing a mycelia-containing agar block on a hydrophobic surface. The results were captured at 24, 48, and 72 h post inoculation (hpi). The HDA formation rate after 24 h was measured by counting the appressoria formed on the hyphal tip (*n* > 100). **(B)** Lipid droplets in the hyphal tip were stained with Nile red. The fluorescence intensity was measured in gray scale. The data presented in panels **(A,B)** were analyzed using Scheffe’s test (*p* < 0.05). The scale bar represents 10 μm. **(C)** The HDA-governing genes *MoLRG1* and *MoMSN2* were up-regulated in Δ*Momyb1* relative to the WT and *Momyb1c* on mycelia grown on an artificial hydrophobic surface. **(D)** The HDA-associated genes *CUT2*, *MOJMJ1*, *MoMSB2*, *MST7*, *MST11 PMK1*, *MoSHO1*, and *MoHOX7* are differentially expressed in Δ*Momyb1* compared to the WT when grown on a hydrophobic surface. Error bars (± SDs) were calculated using 2^–ΔΔCt^, and β*-tubulin* was used for normalization. **(C,D)** Statistical significance was determined by one-way analysis of variance (ANOVA), *p < 0.05, **p < 0.01, ***p < 0.001, different letters indicate significant differences, *p* < 0.05. Values are means of three biological replicates.

The integrity of the mycelial cell surface is critical to proper HDA development (Talbot et al., 1996). Thus, we analyzed HDA formation in Δ*Momyb1* on various hydrophobic surfaces. On glass cover slips, the WT and *Momyb1c* formed HDA after 24 h, whereas Δ*Momyb1* did not initiate HDA formation during the period of observation (Figure 5A). This outcome demonstrates that MoMYB1 is necessary for HDA formation on hard, hydrophobic surfaces.

The accumulation of lipid droplets from the hyphae into an incipient appressorium is a distinctive characteristic of HDA formation (Kim et al., 2009). Subsequently, in the mature appressorium, lipid droplets are degraded in the course of turgor generation. To understand the lack of HDA in Δ*Momyb1*, we performed Nile red staining followed by epifluorescence microscopy for observation of the subcellular distribution of lipid droplets in the nascent HDA. Lipid droplets were arranged and accumulated in clusters located predominantly in the emerging HDA at the hyphal tip in the WT and *Momyb1c* (Figure 5B). However, in Δ*Momyb1*, the lipid droplets were distributed equally, unfused within the hyphae, and not concentrated near the hyphal tip for the initiation of HDA genesis. Peroxisomes contribute to the distribution of lipid droplets and enforce their polar accumulation (Goh et al., 2011). In accordance with our previous results, we observed reduced accumulation of the red fluorescent protein (RFP)-tagged peroxisome marker RFP-SKL (Goh et al., 2011) in the nascent HDA of Δ*Momyb1* relative to that of the WT (Supplementary Figure 4). This result suggests that the membrane trafficking system, which releases secretory vesicles, is compromised in Δ*Momyb1*. Several genes, including *MoLRG1* and *MoMSN2*, were reported to be essential for HDA formation, and their deletion impaired HDA genesis. Surprisingly, the transcript levels of these HDA-associated genes were consistently elevated in Δ*Momyb1* compared with the WT and *Momyb1c* (Figure 5C). This result suggests that MoMYB1 is a transcriptional regulator of *MoLRG1* and *MoMSN2* that regulates HDA formation. To substantiate this possibility, we analyzed the transcriptional activation of additional genes that are essential for HDA formation. We analyzed the expression of *CUT2*, *MOJMJ1*, *MoMSB2*, *MST7*, *MST11*, *PMK1*, *MoSHO1*, and *MoHOX7* in the WT and the Δ*Momyb1* strain grown on a hydrophobic surface. Consistently, the transcript levels of these HDA-associated genes were elevated in Δ*Momyb1* compared with the WT based on three biological replicates (Figure 5D). In conclusion, the consistent up-regulation of HDA-associated genes in Δ*Momyb1* indicates that MoMYB1 is a transcriptional repressor of their expression. The consistent up-regulation of HDA-associated genes and the concurrent lack of HDA formation suggest an underlying fine-tuned transcriptional process to establish a precise protein abundance of these factors to enable HDA formation.

### Distinct Hypha-Driven Appressorium Formation Processes on Host and Non-Host Plant Surfaces

**FIGURE 6:**
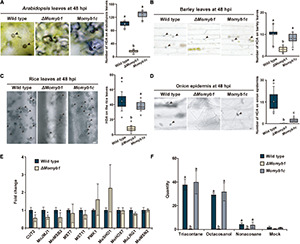
MoMYB1 is involved in host plant recognition accompanied by the modulation of factors that determine HDA formation on host plants. **(A–D)** HDA formation was tested on leaves of **(A)** *Arabidopsis thaliana*, **(B)** rice, **(C)** and barley, and on **(D)** onion epidermis, at 48 hpi. Black arrowheads indicate HDA. Significance was analyzed using Scheffe’s test (*p* < 0.05). All experiments were performed with three biological replicates. The scale bar represents 10 μm. **(E)** Expression of the HDA-associated genes *CUT2*, *MOJMJ1*, *MoMSB2*, *MST7*, *MST11, PMK1*, *MoSHO1*, *MoHOX7*, *MoLRG1*, and *MoMSN2* in Δ*Momyb1* compared to the WT grown on rice leaves. **(F)** Analysis of the effects of the epicuticular wax components triacontane, octacosanol, and non-acosane on HDA formation in the WT, Δ*Momyb1*, and *Momyb1c* at 24 h post-application. **(E,F)** Error bars indicate the standard error of the mean (± SEM); statistical significance was determined by one-way ANOVA against the mock/WT control, *p < 0.05. Letters above bars represent significance groups, *p* < 0.05. Values are means of three biological replicates.

To further assess the ability of Δ*Momyb1* to form HDA on host and non-host plant surfaces, we performed mycelia-based agar block infections on the host plants *Oryza sativa*, *Arabidopsis thaliana* (Park et al., 2009), and *Hordeum vulgare* and on the non-host plant *Allium cepa* (onion). Intriguingly, we detected a small amount of HDA in Δ*Momyb1* on *Oryza*, *Arabidopsis*, and *Hordeum* leaf surfaces compared to significantly higher abundances in WT and *Momyb1c* (Figures 6A–C). However, on the epidermis of the non-host onion, Δ*Momyb1* failed to generate HDA (Figure 6D), similar to the lack of HDA observed on an artificial hydrophobic surface (Figure 5A). These results demonstrate that MoMYB1 is involved in the perception of hydrophobic surfaces, as observed on the cover slip and onion epidermis. However, MoMYB1 is of minor importance in the triggering of HDA formation on host plant surfaces, suggesting that HAMP perception triggers a different mechanism underlying HDA formation. Thus, we conclude that the aerial mycelium in Δ*Momyb1* is particularly compromised in its perception of hard, hydrophobic surfaces, but remains capable of perceiving HAMPs, which partially complements the Δ*Momyb1* phenotype.

To test whether MoMYB1 is involved in the perception of epicuticular HAMPs, we applied various alkanes and primary fatty alcohols that are common in the wax layer of plants. Non-acosane and triacontane are straight-chain alkanes with 29 and 30 carbon atoms, respectively, and octacosanol is a straight-chain aliphatic 28-carbon primary fatty alcohol. These HAMPs were dissolved in chloroform (mock) and administered to the WT, Δ*Momyb1*, and *Momyb1c*. All three substances triggered HDA formation in the WT and *Momyb1c*, with no such formation observed in the mock-treated samples (Figure 6F). However, in Δ*Momyb1*, we observed no HDA formation after HAMP application. This finding indicates that, in contrast to other HAMPs, MoMYB1 is required for the perception of these epicuticular wax components that provoke HDA generation. Plant wax components, such as long-chained primary alcohols and alkanes, make the plant surface hydrophobic. Thus, the absence of MoMYB1 affects the recognition of physical surface conditions.

Appressorium development follows a serial mechanism corresponding to initiation after the recognition of an appropriate surface, penetrating structure formation, and maturation using melanin. Based on our findings, we raised the question whether the expression profiles of HDA-associated genes on a rice leaf surface differs between the WT and Δ*Momyb1*. Genes that were up-regulated on an artificial hydrophobic surface in Δ*Momyb1* were down-regulated (e.g., *CUT2*, *MoJMJ1*, and *MoMSB2*) or showed expression indistinguishable from that of the WT (e.g., *MST7*, *MST11*, *PMK1*, *MoSHO1*, *MoHOX7*, *MoLRG1*, and *MoMSN2*) on the rice leaf (Figure 6E). Our observation of abundant Δ*Momyb1*-derived HDA on the host leaf surface indicates that MoMYB1 determines the degree of host plant recognition, thereby coordinating HDA formation in accordance with fine-tuned control of HDA-associated genes.

### MoMYB1 Is Critical to *Magnaporthe oryzae* Virulence

**FIGURE 7:**
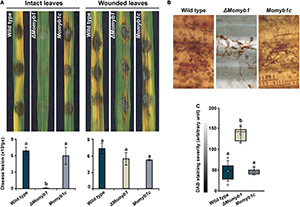
Reduced HDA formation on rice leaves and attenuated fungal virulence in Δ*Momyb1*. **(A)** Intact and wounded rice leaves were inoculated using an agar block containing mycelia of the WT, Δ*Momyb1* and *Momyb1c*. The symptoms were measured 9 days post-infection with ImageJ. Statistical significance was determined by one-way ANOVA against the WT control. Letters above bars represent significance groups, *p* ≤ 0.05. Error bars show ± SEM. **(B,C)** Evaluation of H_2_O_2_ levels through DAB staining 48 h after infection with the WT, Δ*Momyb1* and *Momyb1c.* Outliers are depicted as dots (Min/max range). Statistical significance was determined by one-way ANOVA; different letters indicate significant differences, *p* < 0.01. Representative images of three biological replicates are shown.

Owing to the finding that Δ*Momyb1* shows compromised HDA generation, we investigated whether its virulence and host colonization are affected. To that end, we evaluated pathogenicity by placing mycelia agar blocks of the WT, Δ*Momyb1*, and *Momyb1c* on intact and wounded rice leaves for the assessment of virulence and HDA formation. On wounded leaves, the disease lesion index did not differ significantly among the three strains (Figure 7A), showing that HDA formation is non-essential to infection if the first line of defense, namely the epidermal layer, has already been breached. On intact leaves, however, Δ*Momyb1* exhibited strongly reduced virulence accompanied by weak formation of infection structures and leaf colonization (Figure 7A).

The reduced virulence of Δ*Momyb1* might be mirrored by a diminished plant immune response following Δ*Momyb1* application. *In situ* accumulation of reactive oxygen species (ROS), such as hydrogen peroxide (H_2_O_2_), is one of the first defense reactions after the perception of a biotic threat (Apel and Hirt, 2004; Volz et al., 2018, 2019a, 2019b, 2020). Thus, to assess the *in planta* accumulation of H_2_O_2_, we performed *in situ* 3,3′-diaminobenzidine (DAB) staining of intact leaves inoculated with the WT, Δ*Momyb1*, and *Momyb1c* (Figures 7B,C). WT- and *Momyb1c*-infected plants showed high DAB staining intensity (approximately 50 arbitrary units) and Δ*Momyb1*-inoculated plants showed weak staining (130 arbitrary units). This difference indicates a reduced plant defense response to Δ*Momyb1* infection, which correlates with the reduced abundance of HDA and diminished plant colonization.

### MoMYB8 Determines Cell Wall Integrity and Melanin Biosynthesis

**FIGURE 8:**
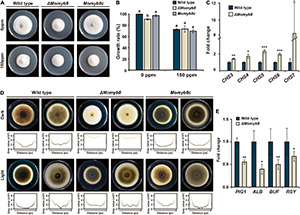
MoMYB8 contributes to cell wall integrity and melanin biosynthesis. **(A)** Growth assay after application of the environmental stress agent CFW to the WT, Δ*Momyb8*, and *Momyb8c.* CFW was administered at 150 ppm, and growth was measured after 9 days. **(B)** The growth inhibition rate of CFW on the WT, Δ*Momyb8*, and *Momyb8c* was determined (diameter of untreated strain - diameter of treated strain)/(diameter of untreated strain × 100). Duncan’s test was performed with three biological replicates (*p* < 0.05). **(C)** Expression patterns of seven *CHS* genes in Δ*Momyb8* are shown. The fold-change values relative to the WT were calculated using 2^–ΔΔCt^, and β*-tubulin* was used for normalization. Error bars (± SDs) represent the range of three biological replicates of qRT-PCR. Student’s *t* test with three biological replicates was performed to determine significance. *p < 0.05, **p < 0.01, ***p < 0.001. **(D)** Each strain was cultured on TCM for 9 days (left) and 10 days (right) at 25°C in the presence or absence of light. Below each image, the plot profile shows melanization intensity from analysis of a gray-scale image. **(E)** Expression patterns of critical genes involved in melanization in the WT and Δ*Momyb8* were analyzed by qRT-PCR. β*-tubulin* was used for normalization, and fold-change values relative to the WT were calculated using 2^– ΔΔ*Ct*^. Error bars (± SDs) represent the range of three biological replicates. Significance was determined using Student’s *t* test (*p < 0.05, **p < 0/01, ***p < 0.001).

To investigate the functional roles of MoMYBs in response to environmental stress, we applied the cell wall stress agent calcofluor white (CFW), which also functions as a chitin-binding agent. We previously observed a reduced vegetative growth phenotype of Δ*Momyb8* (Figure 3D); however, with the use of CFW-supplemented media, we found that Δ*Momyb8* showed weaker mycelial growth retardation than did the WT and *Momyb8c* (Figures 8A,B). Owing to the fact that CFW is a chitin-binding agent, we analyzed chitin synthase (*CHS*) gene expression in Δ*Momyb8* to explore whether MoMYB8 is involved in chitin metabolism. The *CHS* family members 3, 4, 5, 6 and 7 exhibited elevated transcript levels in Δ*Momyb8* compared with those in the WT (Figure 8C). Overall, these results indicate that MoMYB8 plays crucial roles in cell wall integrity and the inhibition of chitin biosynthesis, thereby shaping the potential of fungi to respond to environmental stresses.

Melanin is a major component of the fungal cell wall. We found that the vegetative mycelia of Δ*Momyb8* were weakly melanized compared with those of the WT and *Momyb8c*. Notably, this phenotype of Δ*Momyb8* is confined to mycelia grown in continuous darkness. In contrast, under continuous light, the melanization of Δ*Momyb8* is similar to that in the WT and *Momyb8c* (Figure 8D). To reveal the role of MoMYB8 in the regulation of fungal melanization, the expression levels of several genes involved in that process were analyzed to identify genes with differential expression. We found that the transcript abundances of *PIG1*, *ALB*, *BUF*, and *RSY*, which encoded proteins determine fungal melanization, were lesser in dark-grown Δ*Momyb8* than in the WT (Figure 8E). In summary, these results suggest roles of MoMYB8 in the regulation of cell wall composition and vegetative growth.

## Discussion

Here, we show that the MYB family in *M. oryzae* is characterized by profound structural and functional diversity. MoMYBs include regions of inherently high conservation, designated the DBD, along with disordered non-MYB regions that show high degrees of divergence and variability in their lengths and organization. The functional roles of non-MYB regions have been poorly characterized due to a lack of thorough investigation. Functional annotation has been conducted for non-MYB regions in *Arabidopsis*, which showed regulatory interactions with other proteins that enable nuclear translocation and post-translational modifications such as phosphorylation (Millard et al., 2019). However, we also identified segments of increased conservation in these disordered regions among closely related MoMYBs. Mapping of the non-MYB regions revealed that they are not strictly located at the C-terminus in *M. oryzae*, in contrast to MYBs identified in humans and plants. The non-MYB region is variable, sometimes occurring in front of the MYB domain and occasionally forming long segments between two repeats (i.e., in the subclade containing MoMYB7). In the subclade containing MoMYB8, MoMYB9, and MoMYB10, the general MYB composition appears to be reversed, with the DBD located at the C-terminus after a long disordered region.

The majority of MoMYBs belong to the R2R3 type, which has been shown to enable proper MYB–DNA interaction about the third alpha-helices in both the R2 and R3 repeats in Arabidopsis. MoMYB1 shows high sequence similarity in the third alpha-helices with them of AtMYB73, AtMYB77 and AtMYB118 which suggests comparable DNA-binding ability and target sequences (Supplementary Figures 7A–D; Waterhouse et al., 2018). The putative MoMYB1 downstream targets MoMSN2 and MoLRG1 contain several *cis*-regulatory sequences in the promoter region characteristic for the binding by AtMYB73, AtMYB77 and AtMYB118 (Kelemen et al., 2015). This analysis suggests that MoMYB1 might exert transcriptional control about MoMSN2 and MoLRG1 by the association to these specific binding sites.

Three MoMYBs, namely MoMYB3, MoMYB6 and MoMYB10, contain only single repeats (R3 type). Single repeats can associate with specific *cis*-regulatory sequences in the major groove of DNA. MYBST1 in *Solanum tuberosum* (Baranowskij et al., 1994), REB1 in *Sc* (Ju et al., 1990; Morrow et al., 1993), and ATMYBL2 in *A. thaliana* (Kirik and Bäumlein, 1996) bind to DNA with at least one repeat, suggesting a regulatory role of single-repeat MYB TFs.

Intriguingly, previous studies have shown that two repeats are required for appropriate DNA interaction (Wei et al., 2012; Zhu et al., 2019). Thus, MYBs of the R3 type conceivably act as regulators of the R2R3 MYB type. R3 MYBs might sequester R2R3 MYBs or prevent them from DNA binding, thereby shaping the responses to environmental and developmental stimuli. A Myb Transcription Factor of *An*, FlbD, is involved in both sexual and asexual differentiation. Mutations in the MYB-domain of FlbD affect the DNA binding ability, conidiospore formation and produced naked ascospores without a periderm (Arratia-Quijada et al., 2012). Interestingly, the neurospora *rca-1* gene complements the aspergillus *flbD* sporulation mutant showing conserved MYB-function between different species (Shen et al., 1998).

Appressoria are essential infection structures that penetrate plant cells; they are formed from conidial germ tubes (GDA) or originate from the hyphal tip (HDA). Appressorium development involves the localization and accumulation of hydrophobins at the fungal rodlet layer (Yan and Talbot, 2016). Mutants of the hydrophobin genes *MPG1* (Talbot et al., 1993) and *MHP1* (Kim et al., 2005) show an “easily wettable phenotype” of aerial mycelia reminiscent of that of Δ*Momyb1*. MPG1 has also been reported to regulate GDA (Talbot et al., 1996). However, Δ*Momyb1* showed loss of hydrophobicity and defective HDA formation. Due to the failure of conidium production, Δ*Momyb1* cannot form GDA. Likewise, mutants of *MoLDB1* (Li et al., 2010) and *MoSOM1* (Yan et al., 2011) have defects in conidiation, HDA development, and the integrity of aerial mycelia. Repeated references to these diverse developmental mutant phenotypes in several studies suggest that the rodlet layer and inherent proteins link aerial mycelium formation with conidium production, including the development of GDA and HDA.

HDA formation is considered to be a backup strategy for plant infection in case GDA formation is compromised in *M. oryzae*. To date, the mechanism underlying HDA formation remains poorly understood, and deserves further investigation because *M. oryzae* is one of the most persistent threats to global food security.

We found that Δ*Momyb1* has severe impacts on the structural integrity of aerial mycelia and HDA formation, depending on the type of hydrophobic surface tested. On an artificial surface and non-host plants, Δ*Momyb1* fails to form HDA, indicating that MoMYB1 is a positive regulator of HDA formation. We propose that the reduced structural integrity of Δ*Momyb1* aerial mycelia interferes with the sensing of hydrophobic surfaces and may mislead the fungal sensing system, consequently misdirecting HDA genesis. Indeed, we found that *MoMsn2*, *MoHOX7*, *MoJMJ1*, and *MoLRG1* were up-regulated in Δ*Momyb1* grown on artificial hydrophobic and non-host surfaces. MoMsn2, MoHOX7, MoJMJ1, MoLRG1 and MoRho2 have been reported to be necessary for HDA formation. This result was surprising, as we anticipated a down-regulation of these key genes. However, the consistent up-regulation of all of these genes highlights MoMYB1 as their transcriptional modulator. Furthermore, it is conclusive that HDA formation is not driven by the up-regulation of specific activators *per se*; instead, a MoMYB1-dependent finely balanced titer of these gene products, including the downstream factors that they regulate, might be important for HDA genesis. Feedback regulations of these up-regulated HDA-associated factors might have a negative impact on HDA formation. Thus, the lack of MoMYB1 interferes with the structural integrity of mycelia and may compromise the homeostasis of factors driving HDA formation, which eventually prevents HDA genesis.

In contrast, on host plant surfaces, Δ*Momyb1* formed HDA in low abundance and the expression of HDA-associated genes was distinct from that observed with growth on an artificial hydrophobic surface. Specific HAMPs may partially trigger HDA formation and compensate for the inability of Δ*Momyb1* to perceive a hard and hydrophobic surface. However, the shortage of HDA on host surfaces clearly demonstrates that MoMYB1 enables host plant recognition to a broader extent. The expression of *CUT2*, *MoJMJ1*, and *MoMSB2* is compromised in Δ*Momyb1*. MoJMJ1 is required for HDA formation. CUT2 and MoMSB2 enable the sensing of physical and chemical signals on the host surface, thereby activating the Pmk1 kinase cascade and eventually triggering HDA and GDA formation. Thus, we conclude that the down-regulation of these factors in Δ*Momyb1* hampers host plant surface recognition and results in the failure to activate the Pmk1-kinase cascade. In conclusion, MoMYB1 is necessary for the perception of hard, hydrophobic surfaces that trigger HDA genesis; however, it appears to be of minor relevance for the perception of HAMPs. The rodlet layer of the aerial mycelium in Δ*Momyb1* is capable of perceiving HAMPs, which partially complements the HDA defect in Δ*Momyb1*.

Melanin is an abundant pigment produced throughout the fungal kingdom (Nosanchuk and Casadevall, 2003). A melanized fungal cell wall helps plant pathogenic fungi to resist and endure the plant defense response (Geoghegan et al., 2017). In *M. oryzae*, the pigment is required for appressorium formation and successful infection. Notably, the melanization of the mycelium and that of the appressorium rely on independent mechanisms. In *M. oryzae*, *ALB*, *RSY*, *BUF* (Chumley and Valent, 1990), and *PIG1* (Tsuji et al., 2000) are involved in the biosynthesis of melanin and its intermediate products. The *pig1* mutant, similar to Δ*Momyb8*, showed specific defects in vegetative mycelial melanization. This finding shows that *MoMYB8* and *PIG1* have overlapping functions in mycelial development, and the down-regulation of *PIG1* in Δ*Momyb8* suggests that MoMYB8 is its upstream regulator. The less-melanized phenotype of Δ*Momyb8* occurs under conditions of constant darkness. The effects of various wavelengths on rhythmic pigmentation in *Cercospora kikuchii* were analyzed (Bluhm et al., 2010). Melanization was compromised in *C. kikuchii* under conditions of constant darkness compared with that under light conditions. The melanization defects in Δ*Momyb8* and *C. kikuchii* occurred solely in constant darkness, suggesting a conserved mechanism regulating melanization.

Previous studies in plants have revealed that MYB regulates the biosynthesis of secondary metabolites, including anthocyanin (Park et al., 2008; Zhu et al., 2019), lignin (Legay et al., 2007), and flavonoids (Xu et al., 2014). Chitin is one of the most abundant polysaccharide components localized to the fungal cell wall. Chitin strengthens the fungal cell wall, and its biosynthesis depends on *CHS* genes (Adams, 2004; Kong et al., 2012). Mutation of *CHS* genes led to increased growth after the application of cell wall–disrupting agents (Zhang et al., 2010). The lack of chitin damages the cell wall, interfering with its integrity (Dong et al., 2015). A large number of *CHS* genes are up-regulated in Δ*Momyb8*, suggesting that MoMYB8 is their upstream regulator. The histidine kinase MoSLN1, which activates melanin and chitin synthesis, depends on CHS activity and polyketide biosynthesis (Zhang et al., 2010). The up-regulation of *CHS* genes in Δ*Momyb8* may interfere with MoSLN1 activity, resulting in compromised melanin and chitin metabolism. The functional role of MoMYB8 appears to be comparable to that of its ortholog *GzMyb008* in *Fg* (Son et al., 2011).

In summary, we obtained a multifaceted view of the MoMYB family, which shows a low degree of conservation in accordance with distinct structural and functional features ranging from fungal growth to pathogenicity.

## Data Availability Statement

The original contributions presented in the study are included in the article/Supplementary Material, further inquiries can be directed to the corresponding author/s.

## Author Contributions

SL, RV, and Y-HL conceptualized the research. SL and RV designed, performed, and analyzed the experiments and wrote the original draft. HS generated the phylogenetic tree. WH and Y-HL edited the manuscript. All authors contributed to the article and approved the submitted version.

## Conflict of Interest

The authors declare that the research was conducted in the absence of any commercial or financial relationships that could be construed as a potential conflict of interest.

## Publisher’s Note

All claims expressed in this article are solely those of the authors and do not necessarily represent those of their affiliated organizations, or those of the publisher, the editors and the reviewers. Any product that may be evaluated in this article, or claim that may be made by its manufacturer, is not guaranteed or endorsed by the publisher.

## Footnotes

1
http://genebank.snu.ac.kr

2
http://cfgp.riceblast.snu.ac.kr

3
https://software.broadinstitute.org/morpheus
