# Supplementary material for: Characterization of the MYB Genes Reveals Insights Into Their Evolutionary Conservation, Structural Diversity, and Functional Roles in Magnaporthe oryzae
Source: Front Microbiol. 2021 Nov 26;12:721530. doi: 10.3389/fmicb.2021.721530 (PMC8660761; doi:10.3389/fmicb.2021.721530)
Supplement: Supplementary file 1 [file Table_1.DOCX]

**Supplementary Table 1.** Identified *MYB* TF genes in *M*. *oryzae*.

| Locus number | Gene name | Domain^a^ | Location^b^ | Protein length (aa) |
| --- | --- | --- | --- | --- |
| MGG 06898 | *MYB1* | Myb, Myb-like | Chromosome 1 | 395 |
| MGG_10426 | *MYB2* | Myb, Myb-like | Chromosome 6 | 1276 |
| MGG_08137 | *MYB3* | Myb-like | Chromosome 2 | 509 |
| MGG_01426 | *MYB4* | Myb | Chromosome 2 | 773 |
| MGG_15357 | *MYB5* | Myb, Myb-like | Chromosome 4 | 456 |
| MGG_05945 | *MYB6* | Myb-like | Chromosome 3 | 352 |
| MGG_02746 | *MYB7* | Myb, Myb-like | Chromosome 7 | 619 |
| MGG_01133 | *MYB8* | Myb-like | Chromosome 5 | 390 |
| MGG_05240 | *MYB9* | Myb-like | Chromosome 3 | 250 |
| MGG_05748 | *MYB10* | Myb-like | Chromosome 1 | 305 |

^a^Domains which having on individual MYB TFs were indicated. Domains were predicted via InterPro analysis.

^b^The location of the genes were found on the NCBI genome database.

**Supplementary Table 2.** Previous studied fungal *MYB* TF genes.

| Species | Gene names | *MoMyb* othologs | Locus numbers | Functions | References |
| --- | --- | --- | --- | --- | --- |
| *Magnaporthe oryzae* | *MoMYB1* | *MYB1* | MGG_06898 | VG, CO, CP, PAR, OS | (1) |
| *Aspergillus nidulans* | *FlbD* | *MYB1* | ANID_00279 | CO, CP, SD | (2) |
| *Neurospora crassa* | *rca-1* | *MYB1* | NCU_01312 | CO, VG | (3) |
| *Fusarium graminearum* | *MYT1* | *MYB5* | FGSG_00318 | SD, BS | (4) |
|  | *MYT2* | *MYB6* | FGSG_07546 | SD, VG, CO, PA, SM | (5) |
|  | *MYT3* |  | FGSG_00324 | CO, GM, VG, PA, SM, SD | (6) |
|  | *GzFlbD* |  | FGSG_01915 | VG, PG, SD, CO | (7) |
|  | *GzMyb002* |  | FGSG_00324 | VG, PG, SD, CO, SM, PA, OS |  |
|  | *GzMyb003* |  | FGSG_01274 |  |  |
|  | *GzMyb004* |  | FGSG_01457 |  |  |
|  | *GzMyb006* |  | FGSG_01925 |  |  |
|  | *GzMyb007* | *MYB9* | FGSG_02538 |  |  |
|  | *GzMyb008* | *MYB8* | FGSG_02719 | SD, FC, CW, AC |  |
|  | *GzMyb011* |  | FGSG_06768 |  |  |
|  | *GzMyb012* |  | FGSG_07448 |  |  |
|  | *GzMyb014* | *MYB7* | FGSG_08713 |  |  |
|  | *GzMyb015* | *MYB4* | FGSG_09807 |  |  |
|  | *GzMyb016* | *MYB10* | FGSG_10269 | VG, PG, SD, CO, SM |  |
|  | *GzMyb017* |  | FGSG_12781 | VG, PG, SD, CO, SM, PA |  |
|  | *GzMyb018* |  | FGSG_01167 |  |  |
|  | *GzMyb019* |  | FGSG_01662 |  |  |
| *Saccharomyces cerevisiae* | *BAS1* |  | YKR099W | HIS | (8) |
|  | *CEF1* |  | YMR213W | CC, SP | (9) |
|  | *Reb1* |  | YBR049C | CC, RD | (10) |
| *Schizosaccharomyces pombe* | *Cdc5p* |  | YMR001C | CC, SP | (11) |

Abbreviations used for the analyzed phenotypes: AC, Acidic stress; AP, appressorium formation; BS, Basic stress; CC, Cell cycle; CO, conidiation; CP, conidiophore development; CW, Cell wall stress; FC, fungicide stress; GM, germination; HIS, activation of GCN4-independent HIS4 transcription; NM, nuclear morphology; OS, osmotic stress; PA, pathogenicity on rice leaf; PAR, pathogenicity on rice root; PG, Pigmentation; RD, rDNA metabolism; SD, sexual development; SM, secondary metabolite production; SP, pre-mRNA splicing; VA, viability; VG, vegetative growth.

**Supplementary Table 3.** Phenotype assays of Wild type and its mutants in individual *MoMYB* genes.

| Strain | Vegetative growth (cm)^a^ | Conidiation (x 10^4^ conidia/㎖)^b^ | Conidial germination (%)^c^ | Appressorium formation (%)^d^ | Rice leaf^e^ | Rice root (cm)^f^ |
| --- | --- | --- | --- | --- | --- | --- |
|  |  |  |  |  |  |  |
| Wild type | 6.19±0.09 | 18.41±2.43 | 95.67±0.88 | 94.78±3.20 | 3.83±0.57 | 1.6±0.4 |
| *ΔMomyb1* | 6.62±0.16*^g^ | 0.00±0.00^**^ | ND^h^ | ND | ND | 1.8±0.1 |
| *ΔMomyb3* | 6.19±0.09 | 15.33±3.69 | 96.00±0.33 | 94.67±3.84 | 3.82±0.36 | 1.5±0.9 |
| *ΔMomyb5* | 6.14±0.08 | 14.56±4.05 | 93.89±2.12 | 95.44±0.84 | 3.52±1.08 | 1.3±0.5 |
| *ΔMomyb6* | 6.24±0.07 | 17.19±2.06 | 95.00±1.53 | 96.44±0.38 | 3.48±0.1 | 1.6±0.5 |
| *ΔMomyb7* | 5.88±0.18 | 17.95±2.45 | 95.33±1.53 | 92.67±3.46 | 3.92±0.21 | 1.6±0.4 |
| *ΔMomyb8* | 5.84±0.17* | 18.06±4.86 | 96.44±1.17 | 93.67±2.08 | 3.59±0.53 | 1.2±0.2 |
| *ΔMomyb9* | 6.09±0.40 | 17.95±5.30 | 95.78±1.84 | 92.22±1.84 | 3.83±0.45 | 1.4±0.2 |
| *ΔMomyb10* | 6.16±0.22 | 16.01±2.89 | 95.56±2.04 | 97.44±0.19 | 3.78±0.47 | 1.6±0.5 |

^a^The vegetative growth was measured after being cultured in TCM agar on 9 days.

^b^Conidiation was measured by counting the number of conidia produced.

^c^The percentage of germinated conidia of each strain is shown.

^d^The percentage of germinated conidia that formed an appressorium is presented.

^e^Disease index was applied to the symptoms of infected rice leaves. It was measured 7 days after inoculation.

^f^The length of infected area on roots is shown.

^g^Student's t-test was used to determine statistical significance. Significant differences are indicated using asterisks (**, P<0.01).

^h^ND = not determined.

**Supplementary Table 4.** List of primers used for creating gene disruption constructs and qRT-PCR.

| Primer name | | Sequences (5' - 3') |
| --- | --- | --- |
| *MoMYB1* | UF | GGATGCACTCCCACGTTAC |
|  | UR | GGATTAAGGCTACGGGAGTAAC |
|  | DF | GATTCCCATCAATGAATTCCGTC |
|  | DR | CCCGAACTCTCCTCACC |
|  | NF | CGTTACTTGGTCTCGGCTTGATG |
|  | NR | GTAGTGGTGGCTCAATTCCAAC |
|  | qRTF | CAAAGCGCATCACGAGC |
|  | qRTR | CTCTCACGGCACTGCTTT |
|  | ComF | CCCGTAGCCTTAATCCTTACTGG |
|  | ComR | CTATTTCGCAGAGCCGGGC |
|  | qRTR2 | ATAAGGTCCACCGCGGGCACGGATT |
|  | qRTF2 | CCAGAACCTCAAGCCGTCAAT |
|  | qRTR3 | GGTGCGGTTGTGAAGTGT |
| *MoMYB3* | UF | ACGCTGCAGTTATTGGCCTTTG |
|  | UR | GGAATAACCGCGCTTGTTGGAG |
|  | DF | GCTGTGCGTGAATATTGGATG |
|  | DR | CTTTGGGGGCCTATTTGAC |
|  | NF | GCCCTCAAGTTAGCACACG |
|  | NR | TCCGACACGAATACGCC |
|  | qRTF | CAGCCTCAACTGATCAGAG |
|  | qRTR | CTCTTGTCATTTATCGAAAGGTC |
| *MoMYB5* | UF | GGCGGGAATCTACCGATTG |
|  | UR | TGGTTTGTCGCGGTATTCC |
|  | DF | CGGTTCTTGACACGAAGCGAAC |
|  | DR | GTCTTATGAGGCCCAGC |
|  | NF | CAACGAAGGCAGATGGCTTGCC |
|  | NR | AAACGGCAAGTATCTGTCGTGCGT |
|  | qRTF | GCGCAAGTCCTGGCTATGG |
|  | qRT R2 | AGCTCGCTATCATCCGAGCGTGGGTC |
| *MoMYB6* | UF | ACCGCGGCGTTTAGAATGT |
|  | UR | GCTGGAGAAGTGATCACG |
|  | DF | TTTACTGGCCCGCACC |
|  | DR | ACACGCATCCGATAAAGGTCAT |
|  | NF | GTTGCCGATTTGACATGCCATG |
|  | NR | TGGTGCCACCATATTATTGGG |
|  | qRTF | CTACGTTAGCCACAACCAAC |
|  | qRTR | CTCCACACTGGGTAACCTC |
| *MoMYB7* | UF | CGTTCTAGTCAGGCACAACATG |
|  | UR | CTGCGATGCGAGGAAACC |
|  | DF | TTCTAGATTGGAGGCCGCAAAG |
|  | DR | GCGTAACCAAGAATGGGATCCC |
|  | NF | GAGATAGAGAGGGGGTTGTCC |
|  | NR | GCATTTTAGTCCCAGGTTGTCG |
|  | qRTF | GTGCAGGCAAGGGAAGG |
|  | qRTR | GGCGTTTGCAGGGCTC |
| *MoMYB8* | UF | GACAATGGGCGGGTGGATAGGG |
|  | UR | GGGGTGTTGGAAGGTAGAC |
|  | DF | TCAACACTGTGTGCGGCAG |
|  | DR | GAGTACCTGGGGCGTTAGG |
|  | NF | GCATCGGTTATTCGAATCTTCCG |
|  | NR | TCAGTTCCCGACAAGGGTAGGG |
|  | qRT F2 | CGGCCGCGGCCCCCGCTGCCGG |

**Supplementary Table 4.** Continued

|  | qRT R2 | GCATTGGGCATAGTAGCGCCGGCAG |
| --- | --- | --- |
| *MoMYB9* | UF | TGCCCCAATGGTTATCCTTCTA |
|  | UR | ATCTCCGCCCACTTTCTACTGT |
|  | DF | GCTTTGTTACAGCTGGCC |
|  | DR | CTAGGTAGGGCAAGACAAGG |
|  | NF | CCACTATAGCTGCATTGCGAGC |
|  | NR | CAAGACAAGGTACGCGCAAC |
|  | qRTF | AACTTCGAACAGCTCTGC |
|  | qRTR | CCAAGTGGATGTCCATGC |
| *MoMYB10* | UF | CCTATGCATAGACTGTGGTACG |
|  | UR | GAATTATGATTAGTGGGGGTGGG |
|  | DF | CGATATCCGTCTGTCTAGGGCG |
|  | DR | CATCAGATGAAAGCCAACGAGA |
|  | NF | CTGCAGACTGCGACAAGG |
|  | NR | GTCGACTGTTCGCGTTCA |
|  | qRTF | TCCAAAGAGGAGTGGCAGGACT |
|  | qRTR | CTCCACGTCCCATGGATG |
| *Beta-tubulin* | qRTF | ACAACTTCGTCTTCGGTCAG |
|  | qRTR | GTGATCTGGAAACCCTGGAG |
| *MHP1* | MHP1_qRT_F | AACCTCGACTGTGGCCAGCCCTCAG |
|  | MHP1_qRT_R | AGGACGCAGCAGCGAGCACGCTG |
| *MPG1* | MPG1_qRT_F | CGTTCTCAGCGGCGAGTGCAAG |
|  | MPG1_qRT_R | CAGCACGAAACGGTGTCCGAGCAGA |
| *MoHOX7* | HOX7qF | CGGACGGCTCCAAGATTCTCC |
|  | HOX7qR | CTGCCACGCTTCATGCCAA |
| *MoMSN2* | MSN2qF | ACATTATGGGTGAGGAGGAT |
|  | MSN2qR | CTTGCCGTAAGAGTTGTAGT |
| *MoLRG1* | LRG1qF | AAATGGGCGATTAGGTGTG |
|  | LRG1qR | CTCAGCGACTCAACACTATT |
| *MoJMJ1* | JMJ1qF | ACGGTAAATGTCTCAAGGTGG |
|  | JMJ1qR | AAAGTAGGTCTTCAAGGTTCGG |
| *MoCUT2* | CUT2qF | AGGGGACCCTCATCATCACC |
|  | CUT2qR | CGGGCAGCGATAAAGTCAAC |
| *MoSHO1* | SHO1qF | GGTACTATCTCCGCATCCGC |
|  | SHO1qR | GTCAATGGCATGCGCAACTC |
| *MoPMK1* | PMK1qF | GGAGTACATTCGCTCGCTGC |
|  | PMK1qR | TCCAAGGCGAGATCCGAAG |
| *MoMST7* | MST7qF | TTGATGGAGCGGGATAACCG |
|  | MST7qR | GGTAGGGGTTTCTAACGGGC |
| *MoMSB2* | MSB2qF | CATCCTCCCTGGAGCTACTCTT |
|  | MSB2qR | TTGGTGCTGTTGTTGTCGTTG |
| *MoMST11* | MST11qF | AGCCAAGTGACCAGATGTCG |
|  | MST11qR | CTGCAGGGTTCGGTGTTTTG |
| *CHS1* | CHS1_F | GCC AGG ACT GCC TCT TTC TTC CAG |
|  | CHS1_R | GCG ACG GGC AAT GCA GCA C |
| *CHS2* | CHS2_F | ACT CGG AGG CAC AGA TGC A |
|  | CHS2_R | ATG GTC CTG TCG CTG TAG ATC TC |
| *CHS3* | CHS3_F | CAG GAT CAC GCC TAA CAC GAA C |
|  | CHS3_R | ACT TGA ACC CAG ACA ATG CG |
| *CHS4* | CHS4_F | GGC AGA GCC AGT ACT GGG G |
|  | CHS4_R | CAA GCG TCA GAG TAG TAC TCG |
| *CHS5* | CHS5_F | CTG CGT ATG CCG GGC CCA C |
|  | CHS5_R | GTT GCG CAG GTT GCT CAT GCT CAT C |
| *CHS6* | CHS6_F | CTG GGA GCA CCA CGG GCA TAC C |

**Supplementary Table 4.** Continued

|  | CHS6_R | GGA GGT TGA ACT GTG ATC CAC C |
| --- | --- | --- |
| *CHS7* | CHS7_F | CAC TCG GCC GGG CGA GCT TCT T |
|  | CHS7_R | GAC GCT TGC CTG CTC AGT GCA GG |
| *BUF* | BUF_qF | CAC TGG GAA AGT GAT CGG T |
|  | BUF_qR | TTA CAT GCA AGC ACC GCC |
| *ALB* | ALB_qF | CCC AAC CGC TGG GAC GAG TAC C |
|  | ALB_qR | CAT AGC GCC GTG CAT CAT C |
| *RSY* | RSY_qF | GAG GTA CAA GGA CAC CAC C |
|  | RSY_qR | ACT CGC CCC AGC GGA TGT C |
| *PIG1* | PIG1_qF | CGT TGC GCT CCT TAC CAG |
|  | PIG1_qR | CCG TCG CTG AAG CGT ATG |

**References**

1. Dong Y, Zhao Q, Liu X, Zhang X, Qi Z, Zhang H, Zheng X, Zhang Z. 2015. MoMyb1 is required for asexual development and tissue-specific infection in the rice blast fungus Magnaporthe oryzae. BMC Microbiol 15:1-10.

2. Arratia-Quijada J, Sanchez O, Scazzocchio C, Aguirre J. 2012. FlbD, a Myb transcription factor of Aspergillus nidulans, is uniquely involved in both asexual and sexual differentiation. Eukaryot Cell 11:1132-42.

3. Shen W-C, Wieser J, Adams TH, Ebbole DJ. 1998. The Neurospora rca-1 gene complements an Aspergillus flbD sporulation mutant but has no identifiable role in Neurospora sporulation. Genetics 148:1031-1041.

4. Lin Y, Son H, Lee J, Min K, Choi GJ, Kim JC, Lee YW. 2011. A putative transcription factor MYT1 is required for female fertility in the ascomycete Gibberella zeae. PLoS One 6:e25586.

5. Lin Y, Son H, Min K, Lee J, Choi GJ, Kim JC, Lee YW. 2012. A putative transcription factor MYT2 regulates perithecium size in the ascomycete Gibberella zeae. PLoS One 7:e37859.

6. Kim Y, Kim H, Son H, Choi GJ, Kim JC, Lee YW. 2014. MYT3, a Myb-like transcription factor, affects fungal development and pathogenicity of Fusarium graminearum. PLoS One 9:e94359.

7. Son H, Seo Y-S, Min K, Park AR, Lee J, Jin J-M, Lin Y, Cao P, Hong S-Y, Kim E-K. 2011. A phenome-based functional analysis of transcription factors in the cereal head blight fungus, Fusarium graminearum. PLoS Pathog 7:e1002310.

8. Tice-Baldwin K, Fink GR, Arndt KT. 1989. BAS1 has a Myb motif and activates HIS4 transcription only in combination with BAS2. Science 246:931-935.

9. Ben-Yehuda S, Dix I, Russell CS, McGarvey M, Beggs JD, Kupiec M. 2000. Genetic and physical interactions between factors involved in both cell cycle progression and pre-mRNA splicing in Saccharomyces cerevisiae. Genetics 156:1503-1517.

10. Rodríguez-Sánchez L, Rodríguez-López M, García Z, Tenorio-Gómez M, Schvartzman JB, Krimer DB, Hernández P. 2010. The fission yeast rDNA-binding protein Reb1 regulates G1 phase under nutritional stress. J Cell Sci 124:25-34.

11. Ohi MD, Link AJ, Ren L, Jennings JL, McDonald WH, Gould KL. 2002. Proteomics analysis reveals stable multiprotein complexes in both fission and budding yeasts containing Myb-related Cdc5p/Cef1p, novel pre-mRNA splicing factors, and snRNAs. Mol Cell Biol 22:2011-2024.
