# Supplementary material for: Characterization of the MYB Genes Reveals Insights Into Their Evolutionary Conservation, Structural Diversity, and Functional Roles in Magnaporthe oryzae
Source: Front Microbiol. 2021 Nov 26;12:721530. doi: 10.3389/fmicb.2021.721530 (PMC8660761; doi:10.3389/fmicb.2021.721530)
Supplement: Supplementary file 2 [file Data_Sheet_1.PDF]

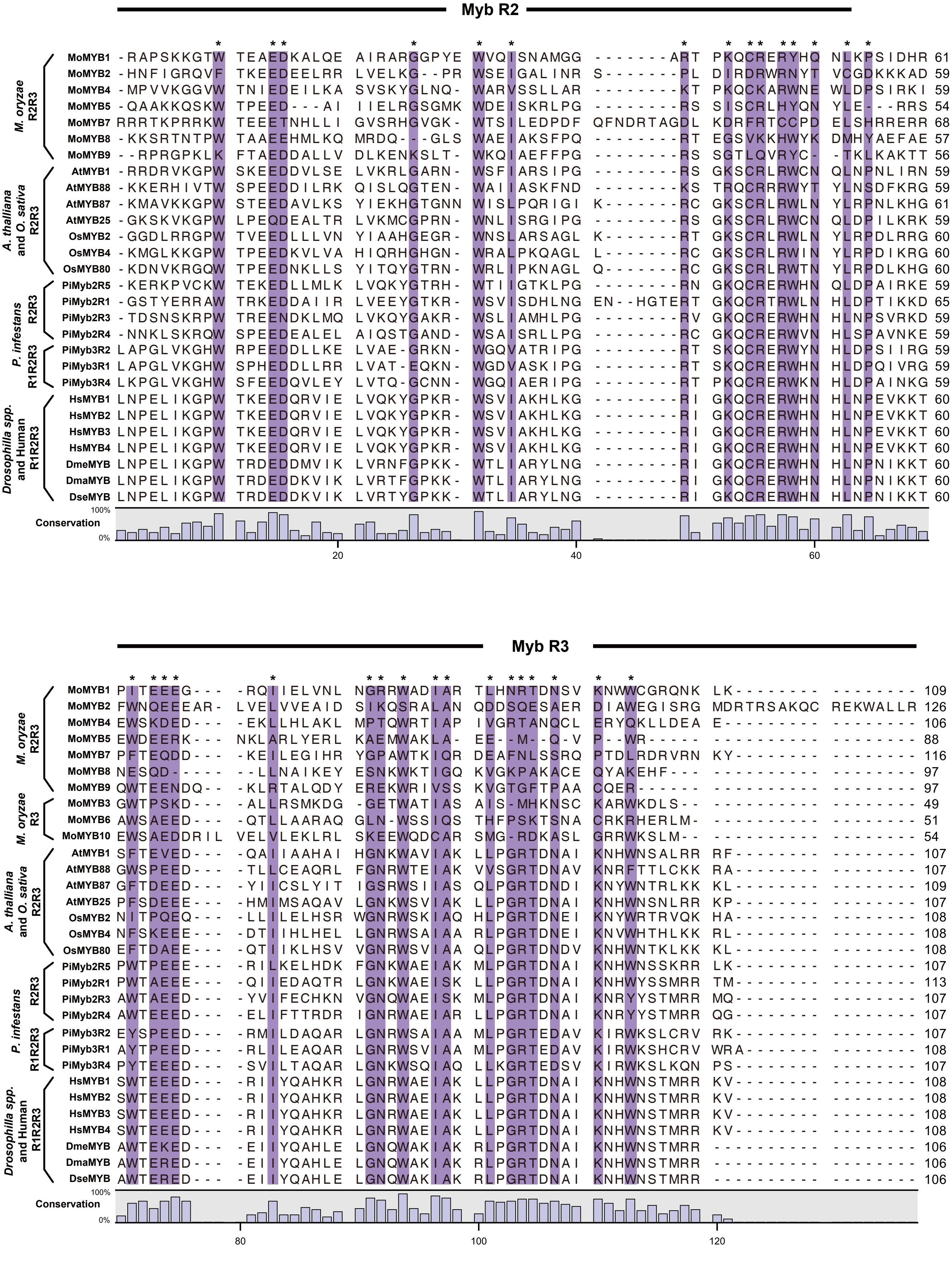

Supplementary Figure 1

MYB-Alignment of the R1R2R3-MYBs from *M. oryzae*, Human and *Drosophila* is depicted. *Arabidopsis thaliana* and *Oryza sativa* R2R3-MYBs, and *Phytophthora infestans* for both repeat domains were aligned. Purple box and asterisks indicate consensus sequence.

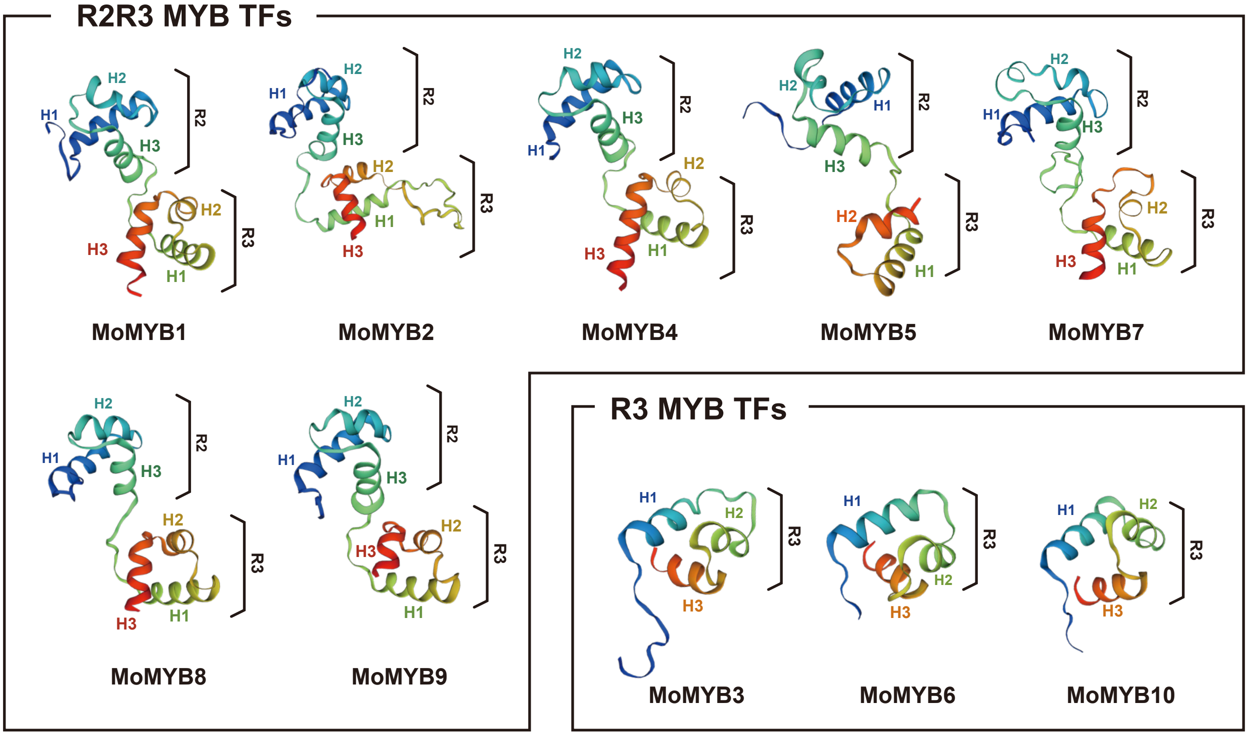

Supplementary Figure 2

Secondary structures were predicted via SWISS-MODEL. MYB TFs in *M. oryzae* are composed either of R2R3- or R3-MYBs.

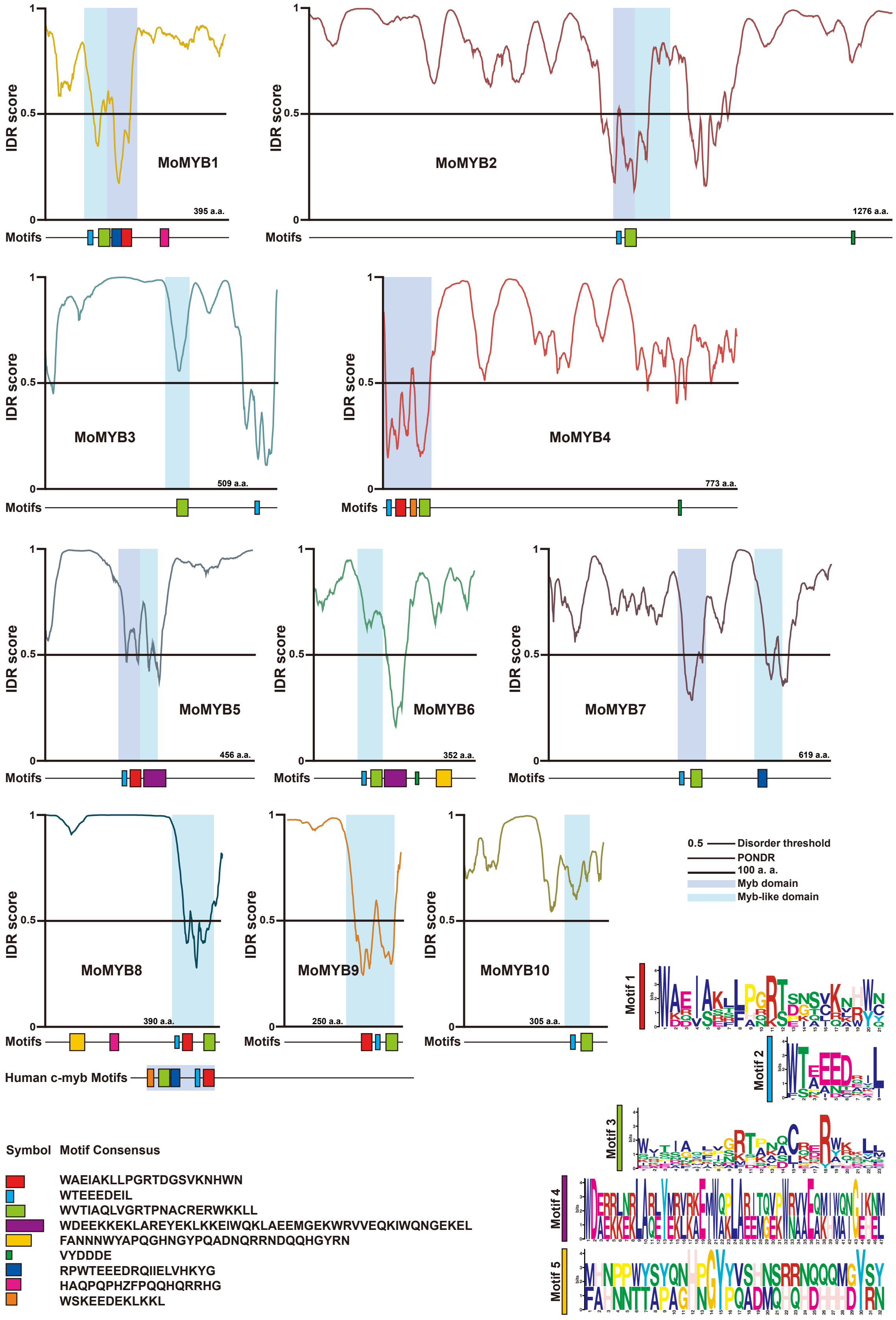

Supplementary Figure 3

Disordered regions were analyzed by the use of PONDR. Motif analysis was conducted by MEME. The disorder threshold is 0.5. The MYB-related protein structures were predicted via SWISS-MODEL.

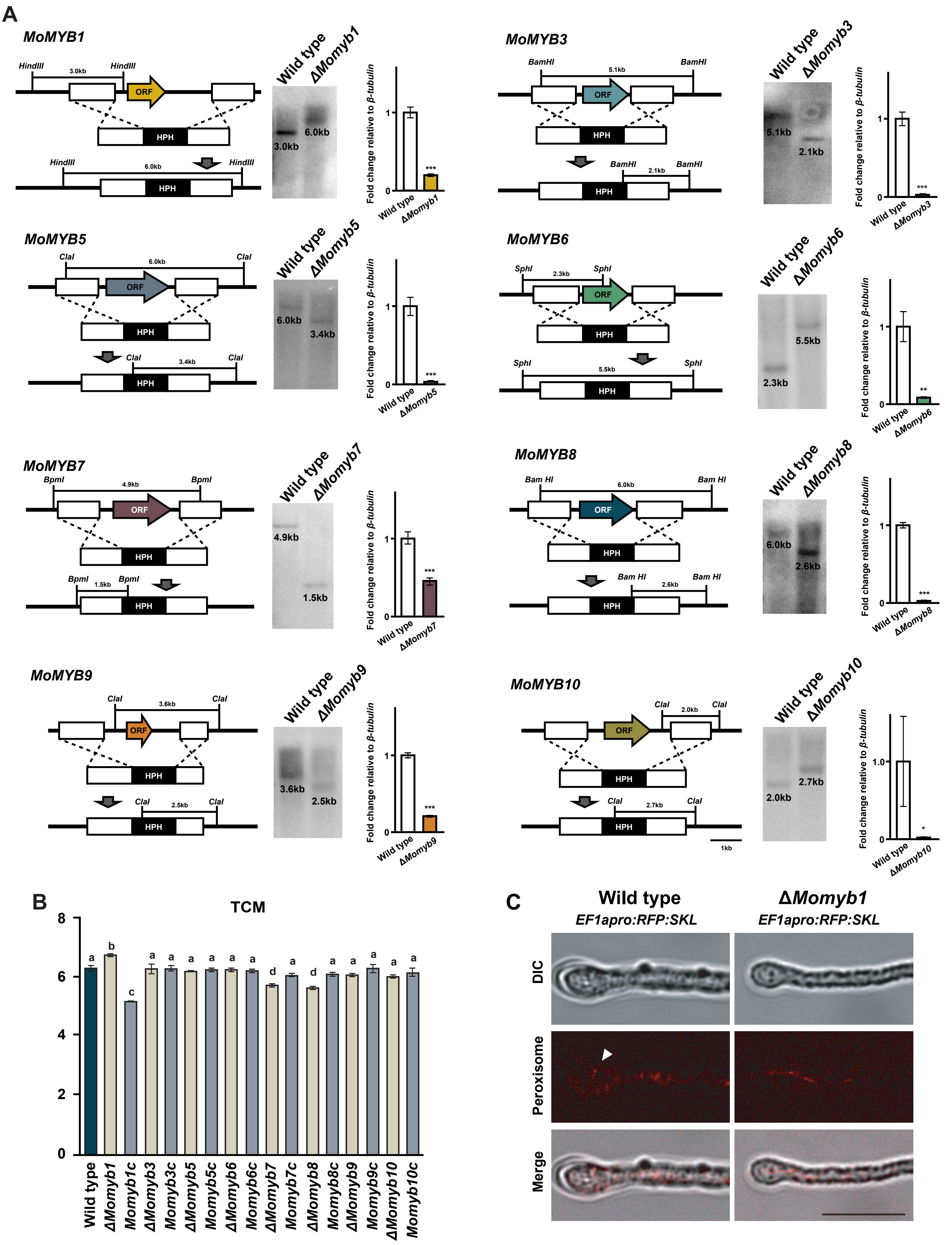

Supplementary Figure 4

MoMYB deletion mutants were generated and their growth, and localization of peroxisomes in *ΔMomyb1* were tested. (A) Gene replacement strategy and southern blot analysis are indicated. Abbreviations are represented as follows: HPH, the hygromycin resistant gene cassette; ORF, Open reading frame. qRT-PCR was performed to validate gene deletion. The fold change values were calculated by $2^{-\Delta\Delta Ct}$, *β-tubulin* gene was used for normalization. Error bars were presented as ±SD from three independent replicates. (B) Growth analysis on TCM agar. (C) Localization of peroxisomes, by the use of the SKL-tagged red fluorescent protein, in wild type and *ΔMomyb1* is depicted. Scale bar indicates 10 μm.

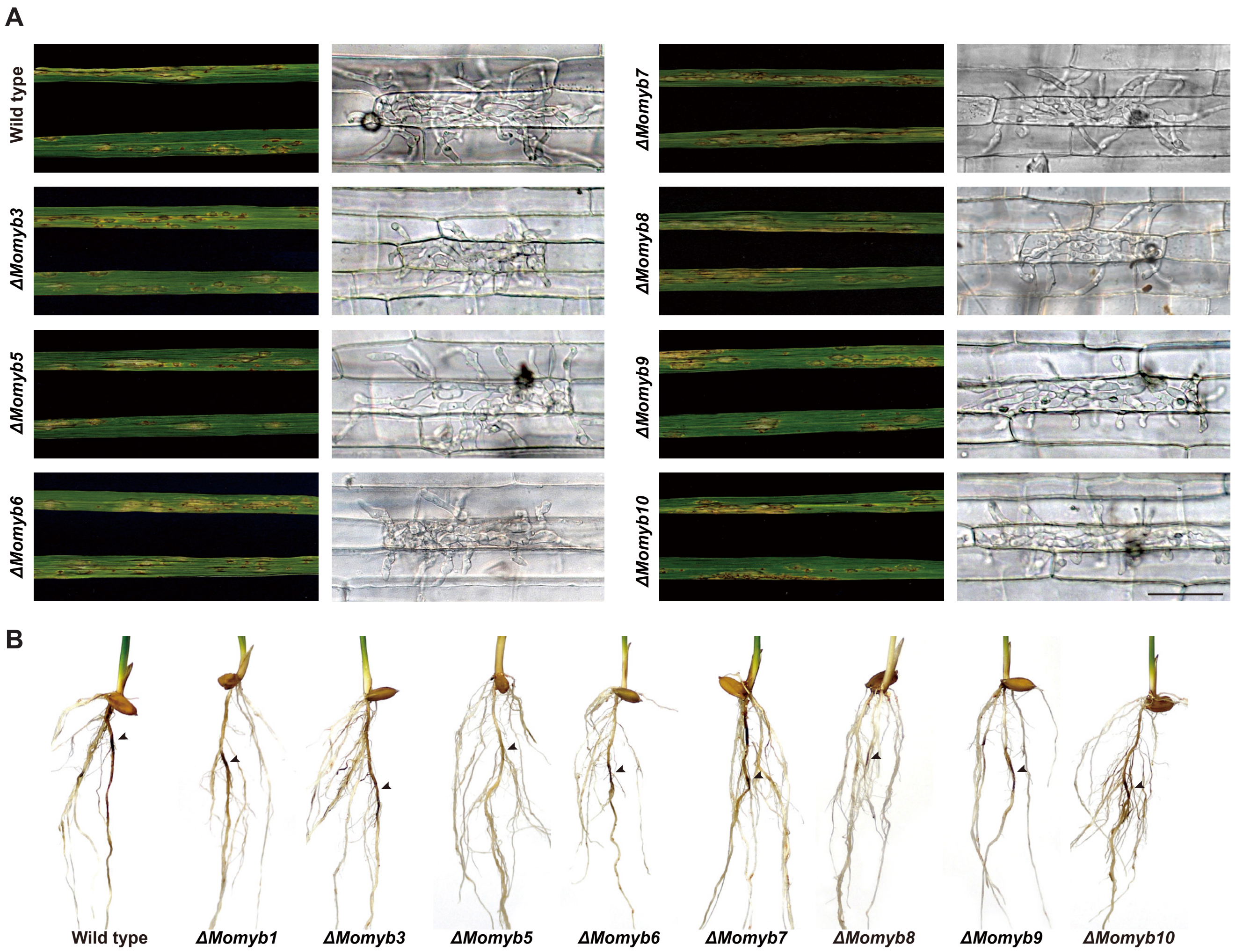

Supplementary Figure 5
Pathogenicity on rice leaves, sheath, and roots. (A) Disease symptoms on rice leaves (7dpi), and sheaths (48 hpi). The scale bar refers to 50 µm. (B) Rice roots were inoculated using mycelial agar block, and resulting symptoms at 14 days after inoculation are shown.

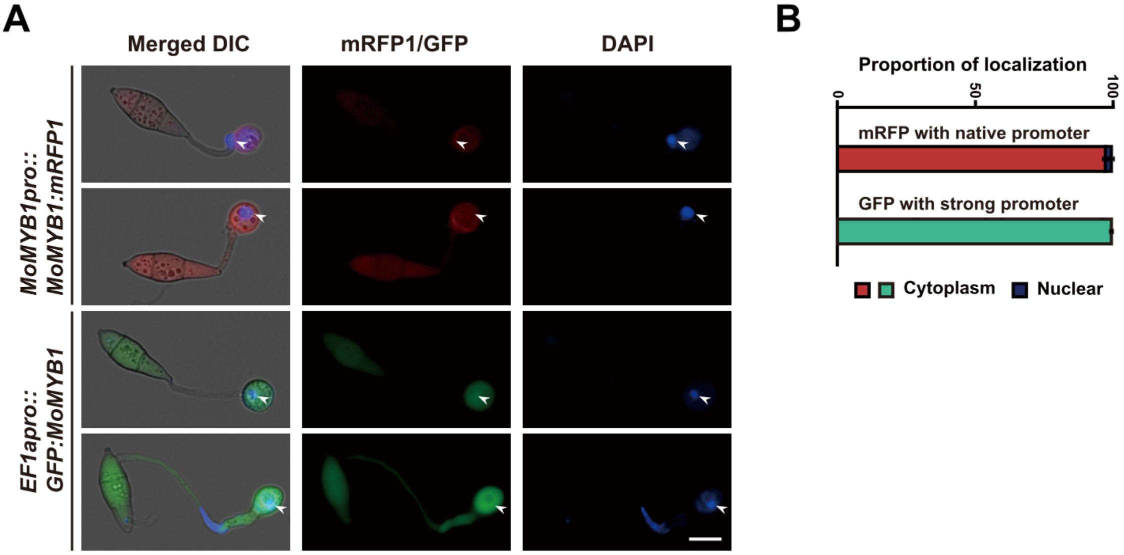

Supplementary Figure 6

Localization of MoMYB1. (A) Subcellular-localization study of MoMYB1 driven by the native and a constitutively active promoter (*EF1apro*). 4′,6-diamidino-2-phenylindole (DAPI) was used for the co-localization study in the nuclei. The quantitative analysis of the MoMYB1 localization is depicted in (B). Scale bars indicate 10μm.

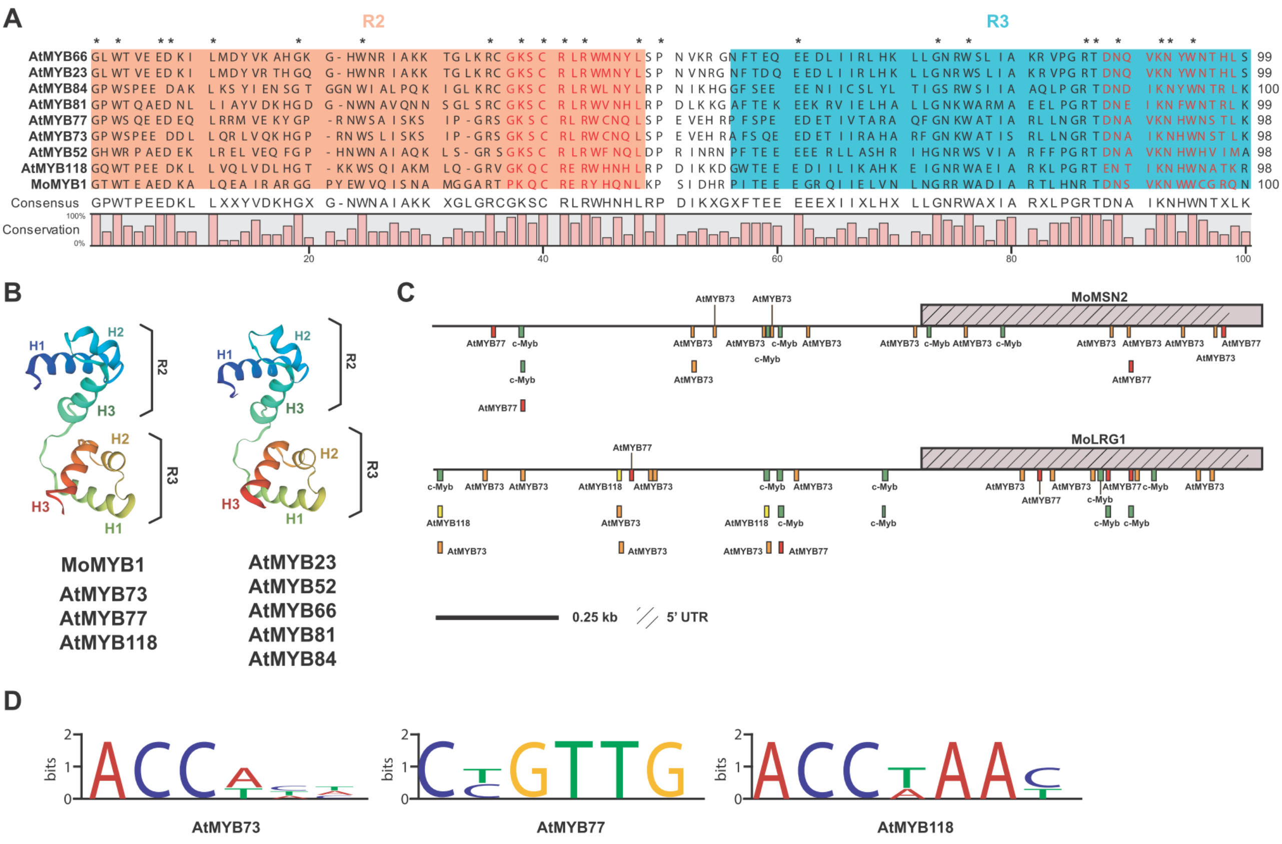

Supplementary Figure 7

Putative DNA binding sites in the 5'- upstream sequence of MoMSN2 and MoLRG1. (A) Sequence alignment of the R2R3 regions of MoMYB1, AtMYB23, 52, 66, 73, 77, 81, 84, 118. Red-colored sequences indicate the third helix of each repeat region, necessary for DNA binding. Asterisks show conserved amino acids among the different MYBs. (B) Predicted secondary structures. (C) Identified targeted cis-regulatory binding sites of AtMYB73, 77, 118, and c-MYB in the promoter region of *MoMSN2* and *MoLRG1*. (D) Binding motifs of AtMYB73, AtMYB77, and AtMYB118 (Kelemen et al., 2015).
